# Phenotypical Analysis of the *Lactobacillus rhamnosus* GG Fimbrial *spaFED* Operon: Surface Expression and Functional Characterization of Recombinant SpaFED Pili in *Lactococcus lactis*

**DOI:** 10.1371/journal.pone.0113922

**Published:** 2014-11-21

**Authors:** Johanna Rintahaka, Xia Yu, Ravi Kant, Airi Palva, Ingemar von Ossowski

**Affiliations:** Department of Veterinary Biosciences, Faculty of Veterinary Medicine, University of Helsinki, Helsinki, Finland; University of Kansas Medical Center, United States of America

## Abstract

A noticeable genomic feature of many piliated Gram-positive bacterial species is the presence of more than one pilus-encoding operon. Paradigmatically, the gut-adapted *Lactobacillus rhamnosus* GG strain contains two different fimbrial operons in its genome. However, whereas one of these operons (called *spaCBA*) is encoding for the functionally mucus-/collagen-binding SpaCBA pilus, for the other operon (called *spaFED*) any native expression of the SpaFED-called pili is still the subject of some uncertainty. Irrespective of such considerations, we decided it would be of relevance or interest to decipher the gross structure of this pilus type, and as well assess its functional capabilities for cellular adhesion and immunostimulation. For this, and by following the approach we had used previously to explicate the immuno-properties of SpaCBA pili, we constructed nisin-inducible expression clones producing either wild-type or SpaF pilin-deleted surface-assembled *L. rhamnosus* GG SpaFED pili on *Lactococcus lactis* cells. Using these piliated lactococcal constructs, we found that the pilin-polymerized architecture of a recombinant-produced SpaFED pilus coincides with sequence-based functional predictions of the related pilins, and in fact is prototypical of those other sortase-dependent pilus-like structures thus far characterized for piliated Gram-positive bacteria. Moreover, we confirmed that among the different pilin subunits encompassing *spaFED* operon-encoded pili, the SpaF pilin is a main adhesion determinant, and when present in the assembled structure can mediate pilus binding to mucus, certain extracellular matrix proteins, and different gut epithelial cell lines. However, somewhat unexpectedly, when recombinant SpaFED pili are surface-attached, we found that they could not potentiate the existing lactococcal cell-induced immune responses so elicited from intestinal- and immune-related cells, but rather instead, they could dampen them. Accordingly, we have now provided the first phenotypical description of a *spaFED* pilus operon, and with that furthered the functional understanding of surface piliation for a particular gut-commensalic genre of piliated Gram-positive bacteria.

## Introduction

Gram-positive piliation embodies the sortase-catalyzed covalent assembly of protein subunits (pilins) into long macromolecular structures, so-called pili or fimbriae that, once becoming cell wall-attached, can extend outwardly from the cell surface into the surrounding environment. Characteristically, each individual pilus takes on a three-pilin architecture, where repeating major pilin subunits form a polymerized backbone, added to which are two ancillary minor pilin subunits, one at the tip for adhesion and another at the base for cell wall anchoring. In some instances, a few Gram-positive pilus structures are comprised of only two types of pilin subunits, with the basal pilin then being excluded (for review, see [1], [2]). As reported in much of the earlier literature, the conceived functional role of pili is essentially to facilitate “first-contact” cellular adhesion and here in the context as virulence factors for a variety of pathogenic Gram-positive species. For this reason, these surface appendages are viewed as potential vaccine candidates against Gram-positive pathogens.

Rather recently, however, Gram-positive pili have taken on a more nonthreatening role, and instead are presumed to act as niche-adaptation factors in non-pathogenic lactic acid bacteria (LAB). As the first reported example of this, a group of Belgian scientists had observed elongated pilus-like protrusions in the cells of *Lactobacillus rhamnosus* GG, a much-perceived beneficial gut commensal and so-utilized probiotic [3]. Subsequently thereafter, these cellular surface structures were then confirmed by us and others [4] as representing a sortase-dependent assemblage of three pilin subunits, much like those that are typically found amongst piliated Gram-positive pathogens. Here in this latter work [4], sequence analysis of the *L. rhamnosus* GG genome had ultimately revealed these pili (called SpaCBA) are encoded by the *spaCBA* operon, which itself contains genes for three pilin-proteins (*spaC*, *spaB*, and *spaA*) and one pilin-specific sortase (*srtC1*). As an assembled multi-subunit structure, the SpaCBA pilus backbone is comprised of SpaA pilins, and as well includes the tip SpaC and basal SpaB pilin subunits [4], [5], each of which is a mucoadhesive component [4], [6] and occasionally also found to be scattered along the length of the pilus itself [6]. Functionally, the SpaCBA pilus not only binds to mucus, but also to collagen protein [7] and an intestinal cell line [8], and as well is able to stimulate biofilm growth [8] and trigger various host immune-cell responses [8], [9]. For these aforementioned activities, the SpaC subunit is credited as a key adhesive factor, but as well, its functionality was quite recently shown to be essential for helping elicit various cellular responses in gut epithelial cells, such as generating reactive oxygen species (ROS), activating the extracellular signal-regulated kinase (ERK) and mitogen-activated protein kinase (MAPK) pathways, and protecting against radiation-induced intestinal damage [10]. Of particular interest, recent findings from our own pan-genome study provide suggestive evidence that the *spaCBA* pilus operon is in fact a rare occurrence in the genome of the *L. rhamnosus* species [11]. Accordingly, for any piliated *L. rhamnosus* strains, it is likely that they will have an augmented niche-specific fitness. Speculatively then for *L. rhamnosus* GG, it is regarded that the adhesive properties associated with the SpaCBA pilus will prolong transient host-gut colonization, and by this then help maximize the apparent health benefits being provided by this commensal.

In addition to the *spaCBA* operon, another set of genes for a second type of pilus (called SpaFED) is found in the *L. rhamnosus* GG genome [4]. Here, the so-called *spaFED* operon also encodes for three pilin subunits (*spaF*, *spaE*, and *spaD*) and a single sortase protein (*srtC2*), and each of whose primary structures shows only minimal sequence identity with their *SpaCBA* gene counterparts [4]. It is noteworthy to mention that while the *spaFED* operon is not widespread among LAB, it has so far been found in all sequenced genomes of other *L. rhamnosus* strains as well as the various strains of two taxonomic cousins, *Lactobacillus casei* and *Lactobacillus paracasei* [12]–[14]. However, as of yet, there are no published reports of the SpaFED pilus being produced or visualized on the cell surface of any of these lactobacilli (including *L. rhamnosus* GG [5]), and so it remains only a hypothetical structure. Moreover, at least for the *L. rhamnosus* GG strain, expression of the *spaFED*-related loci appears to contrast with that of the SpaCBA pilus, wherein constitutive expression of the *spaCBA* operon is thought to be controlled by an upstream DNA region that includes an activating insertion sequence (IS) element [15]. As such, the apparent dormancy of the *spaFED* operon might instead be linked to a yet-to-be-discovered and even perhaps exclusive signaling stimulus that then triggers the inducible transcription of the *spaFED* genes, or in fact may simply be due to deletion or corruption of the regulatory sequence controlling constitutively expressed loci [11]. Even so, solely based on primary structure homologies with other pilins one can reasonably foresee that an assembled SpaFED pilus would have a structural makeup that includes SpaD as the backbone pilin, with the ancillary SpaE and SpaF pilin subunits at the pilus base and tip, respectively.

Previously, in our earlier work [6], we had not only established that each of the *L. rhamnosus* GG SpaFED pilins is expressible as a soluble recombinant form, and so indicating the respective genes lead to properly folded proteins, but of these we also could attribute a mucus-binding functionality to the SpaF pilin. Interestingly, while the predicted location of the SpaF pilin at the pilus tip would be compatible with having an adhesive property, its measured ability to bind mucus was nonetheless surprising, given the absence of any domain homology between its primary structure and other recognized mucus-adhesins [6]. In contrast, the mucoadhesive nature we determined previously for the SpaC tip pilin subunit of the SpaCBA pilus was, in effect, consistent with its primary structure sharing some homology with the lectin-type binding domain of the von Willebrand factor [4]. However, on the other hand, the basal SpaB pilin also lacked any sequence similarity with familiar mucus adhesion domains, but even so is able to bind mucus, and through interactions that we had proposed likely involve electrostatic contacts [6].

Although the assembled and functional SpaFED pilus itself has, for the moment, a conjectural status in *L. rhamnosus* GG, other strains, and some closely related lactobacilli species, we considered it of interest to express the fimbrial *spaFED* operon as a recombinant entity for elucidating its gross structural arrangement, and as well for assessing its functional capacity for adhesion and its stimulative ability for immunogenic responsiveness. As a cloning workhorse, the *Lactococcus* species has proven its utility as a recombinant host, having been used to heterologously produce numerous different proteins (for review, see [16], [17]). Thus, in our case, we employed a strategy that we used beforehand when studying the molecular immunogenicity of the SpaCBA pilus [9], and with a food-grade strain of *Lactococcus lactis*, we genetically contrived the recombinant expression of the *L. rhamnosus* GG *spaFED* pilus operon. Here, two nisin-inducible expression clones were constructed to produce wild-type (WT) and SpaF pilin-deleted surface-assembled SpaFED pili on *L. lactis* cells. Accordingly, with our analysis and characterization of these piliated lactococcal constructs, and to the best of our knowledge, the results we present in this study not only represent the first phenotype characterization of the fimbrial *spaFED* operon, but also these new findings will advance further what is already known functionally about surface piliation in gut commensal-probiotic bacteria.

## Results and Discussion

### *In silico* sequence analysis of the upstream region of the fimbrial *spaFED* operon

In a recent study that examined the genetic organization of the clustered loci encoding SpaCBA pili in *L. rhamnosus* GG [15], it was established that within a stretch of DNA sequence lying just upstream from the *spaC* gene there exists the potential constitutive promoter for regulating *spaCBA* operon expression. Here, the relevant regions identified were the pentanucleotide 5′-TTGAA-3′ (−10) and hexanucleotide 5′-TGGTCT-3′ (−35) sequences, but whereas their composition was said to contrast with the canonical −10 and −35 consensus promoter sequences (i.e., TATAAT and TTGACA, respectively), they were not considered the target of an alternative sigma factor [15]. However, unlike the genes for the SpaCBA pilus, those among the fimbrial *spaFED* operon in *L. rhamnosus* GG seem not to undergo constitutive expression and, in fact, might not even be otherwise expressed and are simply dormant loci [5]. On the other hand, for this particular strain, and presumably as well for other lactobacilli also carrying an intact *spaFED* operon, should this set of genes not be silent it is likely that the regulatory mechanism for producing the corresponding pili is based on some other type of transcriptional control, possibly one that can be inducible through a stimulus-responsive promoter. Related to this, earlier work had already investigated whether certain nutritional components as well as cultivation temperature can act as *spaFED* operon-inducing stimuli in *L. rhamnosus* GG, however the effect of these environmental factors when adjusted did not promote detectable SpaFED pilus production in cells [5]. Alternatively, and as yet another possibility, a recent study has described how phase variation of pili in Gram-positive *Streptococcus gallolyticus* is regulated by an attenuation-like mechanism that involves leader peptide-potentiated pilus gene transcription [18]. Speculatively then, an expressed fimbrial *spaFED* operon might operate on a similar basis.

**Figure 1:**
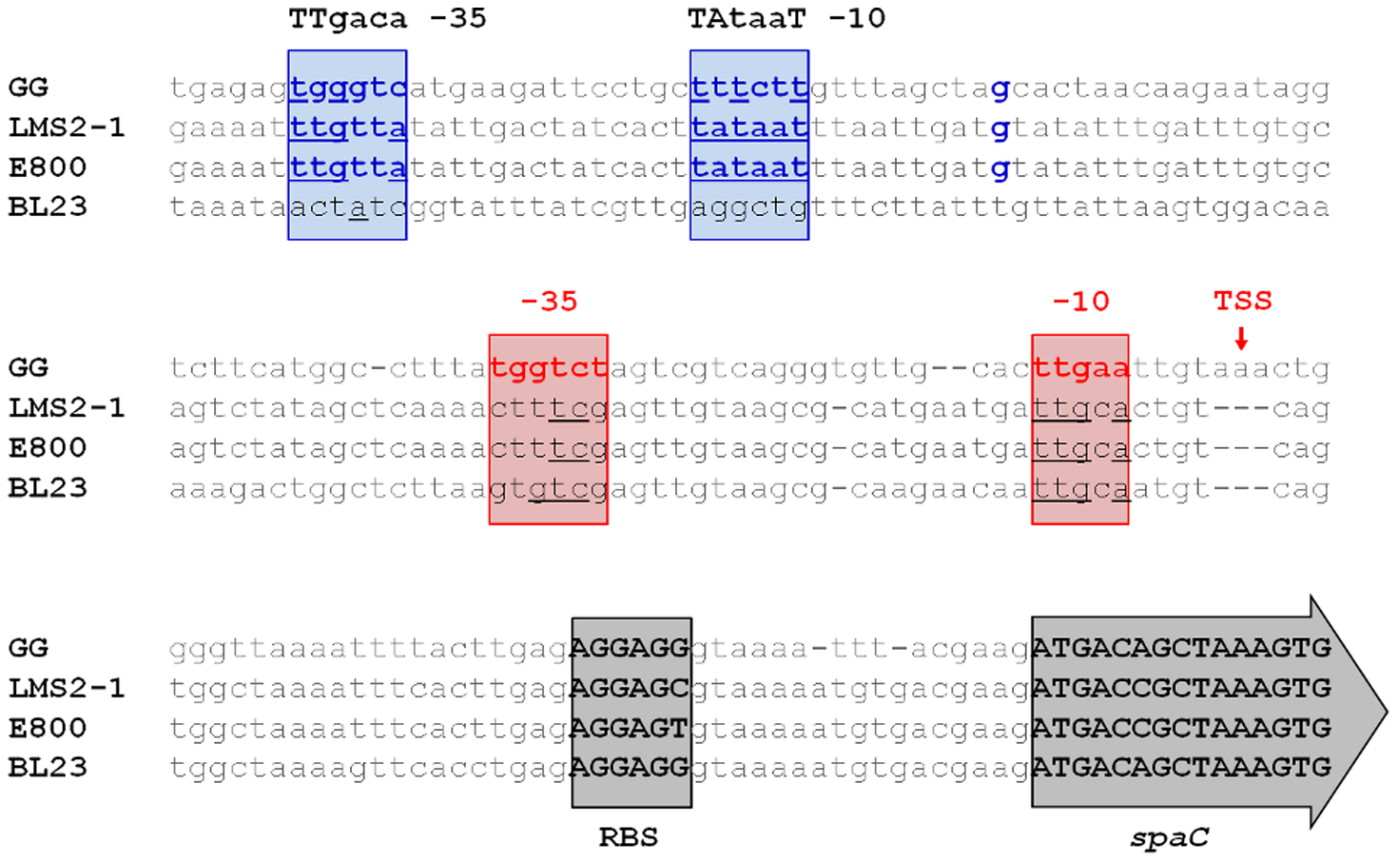
Upstream sequence alignment comparison of the *spaCBA* pilus operon promoter region. Shown is a comparative alignment of an upstream stretch of nucleotide sequence preceding the coding region of the *spaC* pilus gene in the *L. rhamnosus* GG, LMS2-1, and E800 strains, and as well, the *L. casei* BL23 strain. The −10/−35 promoter elements predicted previously for the *L. rhamnosus* GG fimbrial *spaCBA* operon [15] are indicated in red. Nucleotides identical to this consensus region in the LMS2-1, E800, and BL23 strains are underlined. The nucleotide so designated as the transcriptional start site (TSS) for the *L. rhamnosus* GG *spaCBA* pilus locus [15] is indicated. Two hexanucleotide sequences more resembling the typical canonical −10 and −35 consensus promoter elements (as so specified), including a candidate transcriptional initiation nucleotide, are shown in blue. Nucleotides matching the canonical consensus regions are underlined. Nucleotide sequences for the ribosomal binding site (RBS) and the first five codons of the *spaC* gene are in uppercase black boldface lettering.

Because of recent availability of more sequence data for *L. rhamnosus* strains carrying the fimbrial *spaCBA* operon, we decided to first reexamine the earlier results for the SpaCBA pilus promoter region. For this, we made a new comparative analysis that now includes the nucleotide sequences from two other *L. rhamnosus* strains (LMS2-1 [from the NCBI RefSeq database] and E800 [11]), each with the *spaCBA* operon present, but thus far with only the E800 strain having been made known to express surface-assembled pili [11]. In an alignment of upstream sequences for this pilus operon region (Figure 1), we found that for both sequences coming from the LMS2-1 and E800 strains, four out of the five nucleotides comprising the aligned -10 element are an identical match to what had been proposed for *L. rhamnosus* GG [15]. However, for what is perceived to be a similar −35 element in the upstream sequences of LMS2-1 and E800, there are only two matching nucleotides to the analogous region of *L. rhamnosus* GG, but as well with each of the putative sequences still deviating from the normal canonical sequence. Of particular note, the same sort of nucleotide-matching (i.e., 4/5 and 3/6 for the −10 and −35 sequences, respectively) was observed for the equivalent DNA segment in *L. casei* BL23 [19], a strain whose genome encodes a pilus-less phenotype, despite showing evidence of the *spaCBA* operon. However, according to our sequence alignment (Figure 1), and as already observed with the BL23 strain [15], none of the upstream sequences in E800 and LMS2-1 have present a triplet of adenines wherein can be found the corresponding nucleotide designated as the transcription starting point for the *L. rhamnosus* GG *spaCBA*-related loci [15]. Whilst a certain logical consistency exists for the absence of these adenines in the SpaCBA pilus-less BL23 strain, it seems contradictory for these three nucleotides to be missing in the SpaCBA-piliated E800 strain. Thus, we suspect that the so-conceived *spaCBA* operon promoter identified in *L. rhamnosus* GG is most likely not regulating the production of SpaCBA pili in at least the E800 strain. Rather instead, in *L. rhamnosus* E800 (and LMS2-1) a DNA region we found further upstream contains hexanucleotide sequences (Figure 1) that more resemble typical canonical −10/−35 consensus elements, and which in our opinion would better suffice as what regulates a constitutively expressed *spaCBA* pilus operon. Moreover, lying adjacent to the −10 region we also identified a nucleotide that might be taken as the transcriptional start site (see Figure 1). Parenthetically, similar upstream sequence matching such promoter recognition sites can be observed for *L. rhamnosus* GG, but which in the same-aligned DNA segment is less noticeable for the presumably non-piliated *L. casei* BL23 strain (Figure 1).

**Figure 2:**
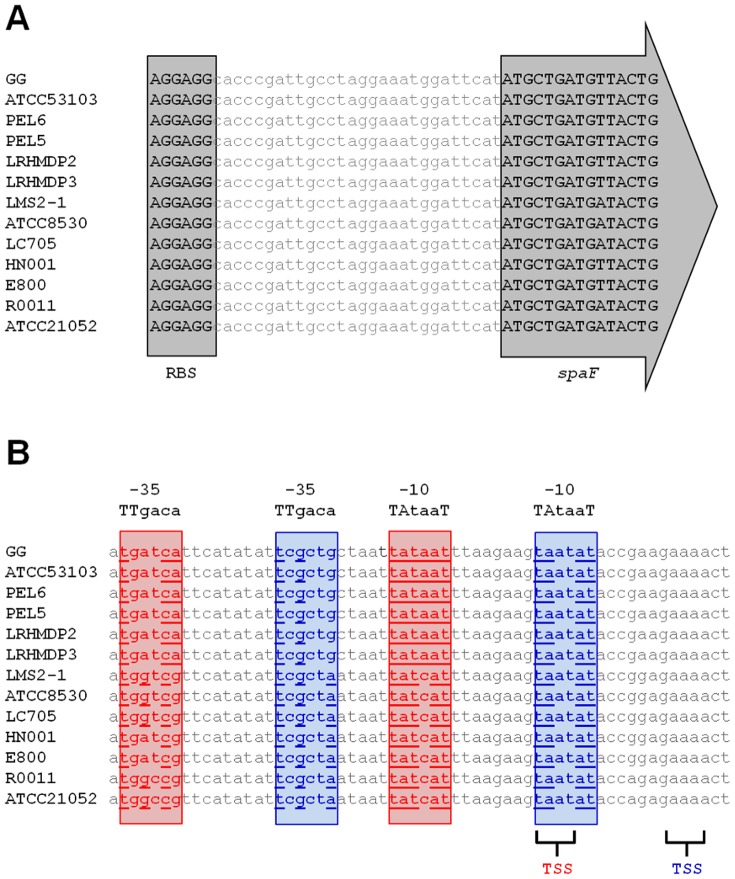
Upstream sequence alignment comparison of the putative promoter region for the fimbrial *spaFED* operon. Segments of DNA sequence are taken from the multiple alignments of *L. rhamnosus* GG, ATCC 53103, PEL6, PEL5, LRHMDP2, LRHMDP3, LMS2-1, ATCC 8530, LC705, HN001, E800, R0011, and ATCC 21052 sequences shown in Figure S1. (**A**) Aligned nucleotides (in uppercase and black boldface type) for the ribosomal binding site (RBS) and the first five codons of the *spaF* locus are indicated. (**B**) Two pairs of hexanucleotide sequences similar to the canonical −10 (5′-TATAAT-3′) and -35 (5′-TTGACA-3′) consensus promoter elements, and as well predicted positions for a transcriptional start site (TSS), are identified in either red or blue. Those nucleotides matching the canonical consensus regions are underlined.

To undertake our *in silico* scrutinization of the “regulatory DNA” for the fimbrial *spaFED* operon, we compiled a multiple sequence alignment encompassing an arbitrarily chosen ∼600-nucleotide (nt) length immediately upstream of the *spaF* locus (Figure S1). For this, we used the corresponding sequence data from the genomes (*n* = 13) of several different *L. rhamnosus* strains that had become available in public databases (either published [20]–[23] or not published) or through our own earlier nucleotide sequencing [11]. Immediately evident from a cursory inspection of this sequence alignment is the minimal variability within the first ∼140-nt segment directly upstream the coding region of the *spaF* gene within the *spaFED* operon. However, then found within the next ∼100-nt stretch further upstream of this is a so-perceived “hotspot” of increased nucleotide variation, and which itself is preceded by a long length of DNA with only a few differences. Though characteristically expected, within close proximity of the two possible methionine-initiation codons (i.e., ATG) in the *spaF* gene, there can be found the nucleotide motif (i.e., 5′-AGGAGG-3′) for a potentially strong ribosomal binding site (RBS), which itself is identical in composition among all of the aligned upstream sequences (Figure 2A). It is rather apparent here that an important prerequisite sequence requirement is retained in the translational unit comprising the *spaF* locus. Moreover, it is also of relevance that similar RBS sequences exist for the remaining loci (*spaE*, *SpaD*, and *srtC2*) of the fimbrial *spaFED* operon (data not shown). With this in mind, since each of the *spaFED*-related genes also encode open reading frames with no premature stop codons, it is also reasonable to expect that unimpeded production of translated pilus proteins would be a possible end result. Accordingly, at the translational level, there is no outright sequence-based evidence to suggest that there be any limiting factors that would potentially preclude the various *spaFED* operon-encoded components from producing an assembled pilus structure, either natively or even recombinantly.

Alternatively, then, and at least amongst those *L. rhamnosus* strains thus far shown to not constitutively produce native SpaFED pili (i.e., GG, LC705, R0011, and E800; data not shown), this perceived failure to do so must instead be related to certain regulatory features at the transcriptional stage. Such a notion is further reinforced by previous reporting that there is no sign of mRNA transcripts for SpaFED pilus loci in *L. rhamnosus* GG, including when cells are grown under varied *in vitro* conditions [5]. Considering this, it would then seem probable that some type of stimulus-responsive promoter controls *spaFED*-related gene expression, but indeed whose very *modus operandi* and inducing signal remain obscure and not so evident to us. However, even with this as a possibility, we found that the typical sequence hallmarks of DNA elements needed for establishing inducible gene expression, such as a symmetric operator site (and target of repressor protein), were not easily noticeable among the aligned sequences (Figure S1). Still though, within the hotspot region so described above we did discern two potential −10/−35 promoter recognition sites, along with candidate nucleotides fitting a possible transcriptional start site (Figure 2B). It should be noted that because *spaFED* loci seem transcriptionally inactive in *L. rhamnosus* GG [5], and so making the corresponding mRNA not readily recoverable, the exact location of the transcription starting point could not unequivocally be confirmed and so remains only a sequence-based prediction. Nonetheless, despite having found what appears suitable canonical promoter regions in the transcriptional unit of the *spaFED* operon, but then without any other sequence attributes of induced (or repressed) gene expression, or for that matter also then lacking the *spaCBA*-like sameness of constitutive pilus expression, one is inclined to also consider other transcriptional scenarios as possible alternatives. Though here our search of this upstream region for key sequence features associated with the attenuation-based regulation of pilus production in *S. gallolyticus* [18], or even for those related to the transcriptional activators that regulate pilus expression in certain Gram-positive pathogens [24], had, in fact, proved less revealing and offered no suggestion that any analogous types of mechanisms are the basis for controlling fimbrial *spaFED* operon expression.

Consequently, it follows that attempting to explain then the puzzling discrepancy between having plausible upstream −10/−35 constitutive promoter sequences and not showing any expression of the SpaFED pilus loci with the accompanying multi-subunit structure would be a conflicting conundrum. As we found no sequence evidence for the occurrence of a transcriptional terminator-like stem-loop lying between the putative promoter region and the beginning of the *spaF* locus (using ARNold [25] at http://rna.igmors.u-psud.fr/toolbox/arnold/index.php), which, having formed, could then prevent *spaFED* operon expression, the inability to produce pili might otherwise seemingly be due to corrupted sequences within this particular DNA region. Accordingly, given the close nucleotide similarity throughout the upstream regions of the various *spaFED* operons (Figure S1), this prohibitory effect on pilus gene expression could be construed as a universal commonality amongst different *L. rhamnosus* strains, but whose molecular reason behind is not entirely clear. Certainly then as a phenotypic outcome, this is at odds with evolutionary-driven genomic modifications that would normally favor achieving some type of fitness benefit. However, until such time that native-expressed SpaFED pilus structures are actually shown to be phenotypically relevant for the *L. rhamnosus* (or *L. casei* and *L. paracasei*) species, it will likely remain rather less understood as to how and why none of the so far examined fimbrial *spaFED* operons exhibit any endogenous transcription and translation activities, but yet have remained genomically persistent and resisted eventual removal through gene loss or decay.

### Surface expression and assembly of recombinant SpaFED pili in *L. lactis*

**Figure 3:**
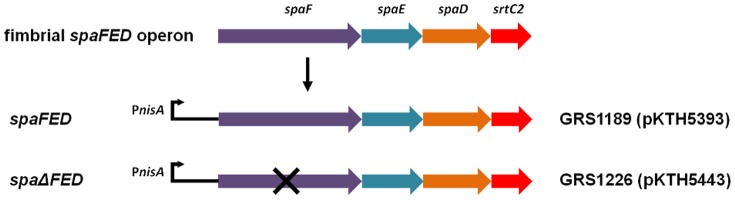
Coding regions of the native fimbrial *spaFED* operon and the corresponding recombinant SpaFED-piliated lactococcal constructs. A schematic representation of the native coding region of the *L. rhamnosus* GG fimbrial *spaFED* operon, including a depiction of the corresponding lactococcal *nisA* promoter (P*nisA*) constructs for expressing WT and SpaF pilin-deleted SpaFED pili, is shown. Names of loci that encode for three different pilin subunits (*spaF*, *spaE*, and *spaD*) and a single pilin-specific sortase (*srtC2*) are given. Deletion of the *spaF* gene is indicated by “×”. WT and SpaF pilin-deleted SpaFED-piliated lactococcal clones (GRS1189 and GRS1226, respectively), and the *spaFED* operon expression plasmids they contain (pKTH5393 and pKTH5443, respectively), are specified.

In our earlier work on *L. rhamnosus* GG piliation, we verified the surface expression and assembly of sortase-dependent *spaCBA* operon-encoded pili through a combination of immunoblotting detection and immuno-electron microscopic analysis, each done using pilin-specific antisera [6], and as well achieved for not only the native structure [4], [5], but similarly for a recombinant form that was produced in *L. lactis* [9]. In an attempt to bring some functional purpose to another type of lactobacillar pilus structure (the so-called SpaFED pilus), though itself still being only conjectural in nature, we applied this same general strategy [9] to characterizing the expression of the *L. rhamnosus* GG fimbrial *spaFED* operon, but then again as a recombinant entity in lactococcal cells. Relatedly, it is worth mentioning that in the *L. lactis* strain IL1403, this bacterium harbors a fimbrial operon (called *pil*) that is also constitutively silent and not expressed under normal growing conditions, although as a recombinant clone, it can produce assembled pili [26]. Nonetheless, in our case, the locus cluster insert containing the coding regions for the three *spaFED* pilus-genes (*spaF*, *spaE*, and *spaD*) and one pilin-specific sortase gene (*srtC2*) was cloned into an expression vector (pKTH5080) carrying a nisin-inducible promoter, with the resulting recombinant plasmid (pKTH5393) then propagated in the *L. lactis* NZ9000 strain. So designated as GRS1189, this recombinant lactococcal construct encodes the expression of WT SpaFED pili. Ancillary to this, we also constructed another *spaFED* operon-containing plasmid (pKTH5443), but which instead encodes for a SpaFED pilus structure lacking the predicted tip-located SpaF pilin subunit (i.e., Δ*spaF*). The corresponding recombinant piliated *Lactococcus* clone was denoted as GRS1226. A schematic representation depicting both SpaFED pilus gene constructs (WT and SpaF pilin-deleted) is shown in Figure 3.

**Figure 4:**
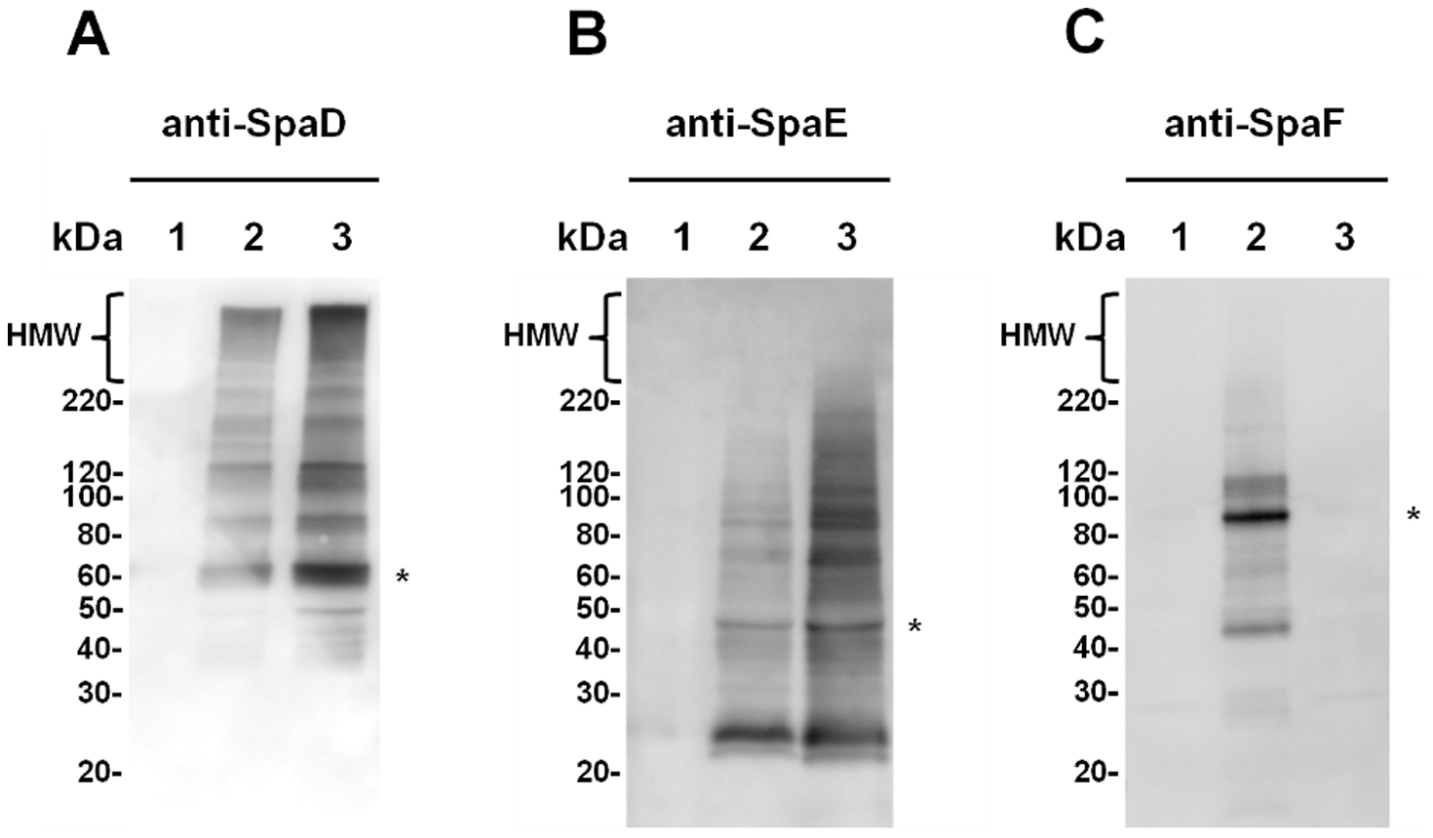
Immunoblot analysis of recombinant SpaFED-piliated lactococcal cells. Immunoblots of lactococcal cells corresponding to the empty vector GRS1052 clone (lane 1) and those to the nisin-induced WT (GRS1189; lane 2) and SpaF pilin-deleted (GRS1126; lane 3) SpaFED-piliated clones were probed separately with polyclonal anti-SpaD (**A**), anti-SpaE (**B**), and anti-SpaF (**C**) sera as described in Materials and Methods. Apparent positioning of monomeric SpaD, SpaE, and SpaF proteins is indicated on the right of each immunoblot by an asterisk. A dense ladder-like smear of high-molecular-weight (HMW) protein bands represents the longest lengths of pili and these are indicated on the top left of the immunoblot. The positions and sizes of the molecular weight markers are shown along the left side of the immunoblot.

One of the most often used detection techniques for demonstrating sortase-dependent pilus protein production involves cellular analysis by immunoblotting with antiserum raised against pilus-subunit protein. Here, various lengths of assembled pili are identified as a high-molecular-weight (HMW) ladder-like smear of compressed protein bands. In this present study, we used the same approach to determine initially whether the major SpaD and minor SpaE and SpaF pilin subunits encoded by *L. rhamnosus* GG *spaFED* loci can be expressed and assembled recombinantly on the surface of lactococcal cells. For this, and post-nisin induction, sonicated whole-cells of WT (GRS1189) and SpaF-deleted (GRS1226) recombinant piliated lactococci were immunoblotted and treated with each of the SpaFED pilin antisera [6] (Figures 4A-C). (The empty vector GRS1052 clone was also included and used as a negative control; see Figures 4A-C, lane 1.) As typically is the case for multi-subunit pili, a clearly distinctive laddered pattern of HMW proteins was detected on the immunoblot of GRS1189 cells when each of the three types of pilin antiserum was used (Figures 4A-C, lane 2). However, the very uppermost HMW bands were not so noticeable with the anti-SpaE and anti-SpaF antisera, but to some extent expected, as fewer numbers of ancillary SpaE and SpaF pilins per pilus would be incorporated, which probably makes them less conspicuous in the lengthier pilus fragments being separated by SDS-PAGE and then not as much recognized when immunoblotted. Also apparent on these immunoblots was the presence of protein bands matching each of the molecular weight sizes for the monomeric SpaD (∼51 kDa), SpaE (∼45 kDa), and SpaF (∼104 kDa) pilin subunits (Figures 4A-C, lane 2). Inferred from these immunoblot data, there is strong suggestive evidence that each of the three pilus proteins (SpaD, SpaE, and SpaF) is a structural constituent of a WT SpaFED pilus, and one which seems not only fully assembled, but surface attached to lactococcal cells as well. Likewise for the GRS1226 (SpaF pilin-deleted) lactococcal clone, the immunoblot data from probing with anti-SpaD and anti-SpaE sera (Figures 4A and B, lane 3) suggests that this construct also expresses pilin-assembled and cell wall-anchored SpaFED pili, but here with the absence of the SpaF pilin in the pilus structure being confirmed when anti-SpaF serum was used (Figure 4C, lane 3). As such, it is interpretable from the immunoblotting results that the fimbrial *spaFED* operon, while not endogenously expressible in *L. rhamnosus* GG, readily underwent transcription and translation in *Lactococcus* cells to produce functioning pilin proteins and an active sortase enzyme that, once having come together catalytically, had formed a recombinant surface-assembled pilus structure.

**Figure 5:**
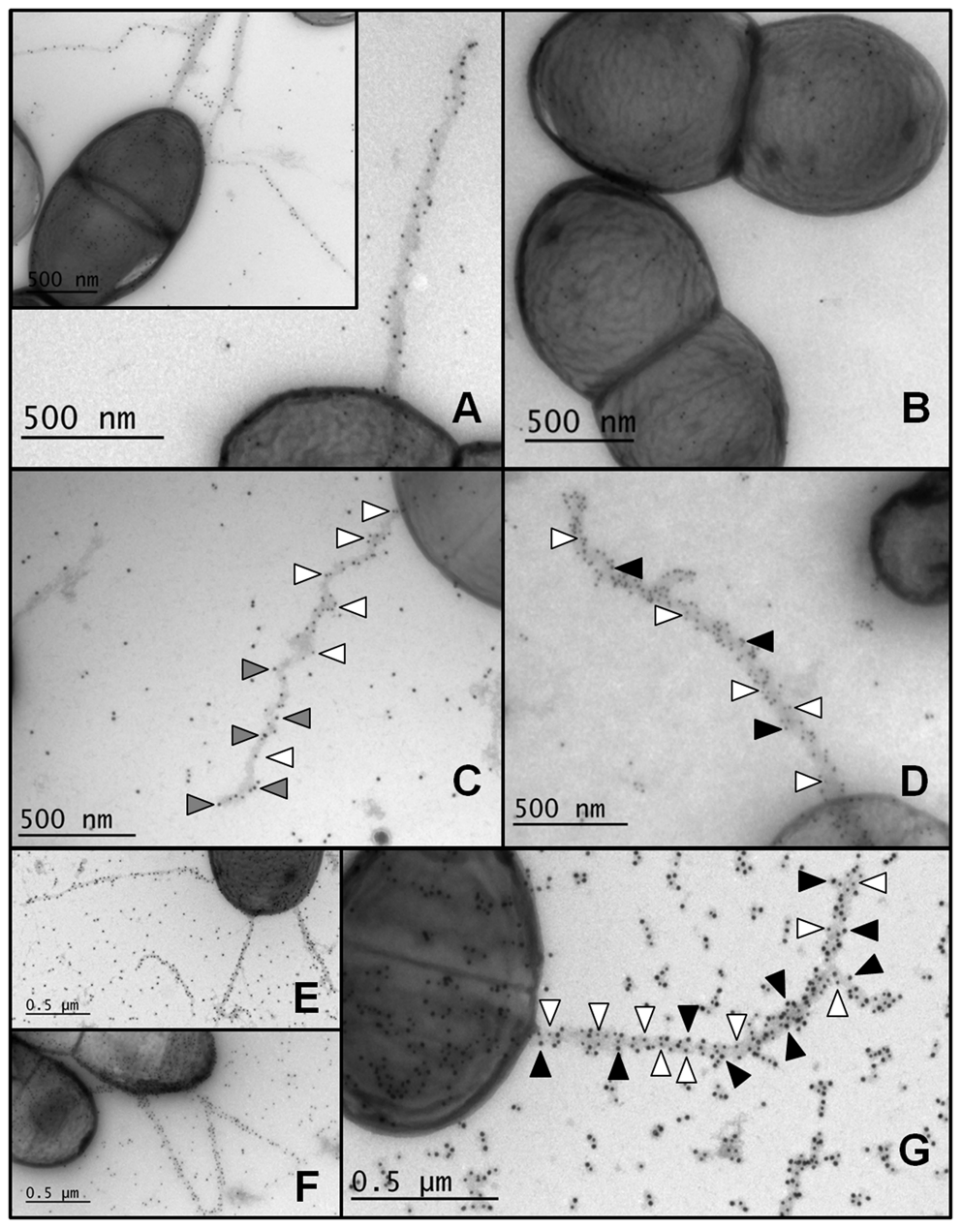
Immuno-electron microscopy of recombinant SpaFED-piliated lactococcal cells. Immunogold pilin protein-labeling and electron microscopic analysis of recombinant WT (GRS1189) and SpaF pilin-deleted (GRS1126) SpaFED-piliated lactococci (including GRS71 cells as a control) were done using the techniques described in Materials and Methods. GRS1189 (**A and inset**) and GRS71 (**B**) cells are single-labeled with anti-SpaD serum and protein A-10-nm gold particles. GRS1189 cells are double-labeled either with SpaD (10-nm; white arrowhead) and SpaF (15-nm; gray arrowhead) antisera (**C**) or with SpaD (10-nm; white arrowhead) and SpaE (15-nm; black arrowhead) antisera (**D**). GRS1226 cells are single-labeled with SpaD antiserum (10-nm) (**E**) as well as double-labeled either with SpaD (10-nm) and SpaF (15-nm) antisera (**F**) or with SpaD (10-nm; white arrowhead) and SpaE (15-nm; black arrowhead) antisera (**G**). A scale bar with dimensions is included in each panel.

As a means to shed some visual light on the architectural arrangement of the individual pilins in the SpaFED pilus structure, we employed the use of immunogold protein labeling and electron microscopy (EM) with both of the recombinant piliated *Lactococcus* clones we had constructed (i.e., GRS1189 and GRS1226). Single-labeling experiments with GRS1189 cells using antiserum specific for SpaD pilin-protein had unmistakably showed the visible presence of pilus-like structures protruding out from the cell wall surface, as evidenced by a length of spherical gold particles (black dots), itself distinguished by an electron-dense region (Figure 5, panel A and inset). As a negative control, a similarly extended assembly of gold particles was not at all obvious when the same SpaD antiserum had been used with the recombinant host GRS71 (*L. lactis* NZ9000) strain (Figure 5, panel B). From these results, it is clear that with anti-SpaD serum the predominant number of gold particles appearing along the pilus length is then representing SpaD pilins, and so in agreement with a foreseen role as the repeating protein subunit that forms the polymeric backbone structure of the SpaFED pilus. In comparison, and through double-labeling experiments using SpaD antiserum in combination with antiserum specific for either SpaF or SpaE pilins, those gold particles identifying the ancillary SpaF and SpaE pilins are seen to be less numerous within the overall SpaFED pilus assemblage (Figure 5, panels C and D), and thus, in effect, this then being a reflection of their predicted structural role. For instance, it is evident that SpaF is indeed the tip-localized pilin in the SpaFED pilus, but in addition a subunit that is also found sometimes deposited along the backbone structure (Figure 5, panel C). Concerning the SpaE pilin, while some gold particles representing this subunit were visibly detected outside of the cell and integrated amongst the SpaD pilins forming the pilus-like protrusions (Figure 5, panel D), we are reasonably certain that the primary structural position of SpaE is that of a basal pilin subunit, and by being buried beneath the cell surface the corresponding gold particles went undetected in our EM experiments. At this point it is worth mentioning that in other types of pili those basal pilins being identified now and then throughout the pilus are just randomly there by chance, as so proposed in the working model of corynebacterial pilus assembly [27], and like what is observed for the ancillary SpaB subunit in the *L. rhamnosus* GG SpaCBA pilus structure [5].

When similar immunogold-labeling experiments were performed with GRS1226 (SpaF-deleted) cells, for the most part we obtained an analogous set of results (Figure 5, panels E-G), but where the SpaF subunit was expectedly missing from the pilus tip, and which was both convincing and visually established when we had done double labeling with anti-SpaD and anti-SpaF sera (Figure 5, panel F). However, elsewhere in the pilus structure there were a few added instances where we could still identify some gold particles for SpaF, this despite our inability to detect SpaF subunits by immunoblotting with anti-SpaF serum (see Figure 5C, lane 3). This result might reflect some cross reactivity of the SpaF antiserum with SpaD pilin-proteins, and then suggesting that those SpaF subunits that are perceived to occur along the WT SpaFED structure (as in GRS1189 cells) are merely an EM artifact. Aptly, this could support an alternative interpretation for the so-postulated “decorative” positioning of the ancillary SpaC pilin in the SpaCBA pilus [4], [5]. Of additional interest, compared to what was observed for the WT SpaFED pilus-expressing GRS1189 lactococci, SpaF-deleted pili in GRS1226 cells were invariably much longer, sometimes more numerous per cell, and more often stretching out into prong-like shapes. As a conceivable explanation for the contrasting visualized manifestations of recombinant WT and SpaF-deleted pili, we suspect that with the deletion of the *spaF* gene from the SpaFED pilus-coding region in GRS1226 lactococci this has lessened some level of burdensomeness on the cellular ability to translate efficiently the remaining pilus loci (i.e., *spaE*, *spaD*, and *srtC2*). As such, this could avail the increased amount of protein that is needed for assembling a lengthier and branched SpaF-deleted pilus structure. Conceivably as well, but more speculatively, because a pilus adhesin is now lacking, this might also mean a self-regulating mechanism that controls pilus biogenesis, one which relies on cell-to-cell feedback signaling using direct contact, is then no longer functional, and so causing pili to be overly elongated than that taken as the “WT norm”.

Also worthy to point out, it was rather noticeable by our EM experiments that for these two recombinant piliated lactococcal clones, they each consisted of a mixture of cell-types with varied numbers of pili per cell, ranging from one to three, but sometimes none at all. Very much akin to our earlier work cloning the SpaCBA pilus [9], we suspect once again that the inherent instability of nisin at physiological pH (and same as the growth medium pH) makes it less effective for triggering uniform protein expression than more stably robust chemical inducing agents. This then could be one possible reason for a mixed population of piliated cells being seen for both the GRS1189 and GRS1226 clones.

### Functional binding attributes of lactococcal-expressed SpaFED pili

**Figure 6:**
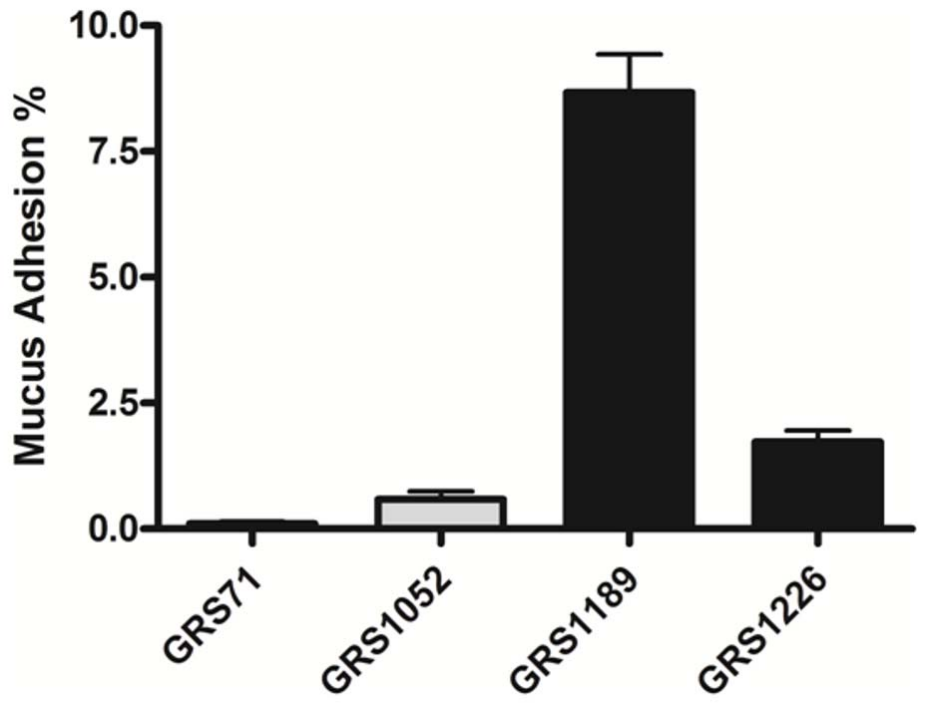
Mucoadhesiveness of recombinant SpaFED-piliated lactococci. *In vitro* mucus-binding assays with normalized (OD600 = 0.5) cultures of recombinant WT (GRS1189) and SpaF pilin-deleted (GRS1126) SpaFED-piliated lactococci, and as well with the vectorless (GRS71) and empty vector (GRS1052) *L. lactis* NZ900 control strains, were carried out as described in Materials and Methods. Triplicate measurements were done for each sample, with three independent experiments having been performed. The standard error of mean (SEM) is shown as error bars. Pairwise differences between the mucus adhesion data for GRS1189 or GRS1226 cells and that for GRS71 cells are deemed extremely significant (*P*≤0.0001).

In our earlier work with recombinant *L. rhamnosus* GG SpaF pilin-protein, we had established its ability to bind to intestinal mucus and largely to the same extent as was shown for recombinant SpaC protein [6]. To confirm whether this binding attribute is also functional in the context of the SpaFED pilus structure, we tested and compared the *in vitro* mucus adhesion capacity of the SpaFED-piliated GRS1189 and GRS1226 lactococcal clones. As shown in Figure 6, nisin-induced GRS1189 cells expressing WT SpaFED pili could noticeably adhere to mucus, and as well, at much the same level as had been reported previously for the recombinant SpaCBA-piliated GRS1185 lactococcal clone [9]. However, quite the opposite was observed with the SpaFED pilus-less GRS71 (vectorless) and GRS1052 (empty vector) lactococcal cells, these being used as negative controls, as they show little, if any, appreciable ability to bind mucus (Figure 6). As one way to pin down whether the SpaF subunit can be attributed to this mucus-binding functionality, we also examined the SpaF-deleted GRS1226 clone and found that the corresponding cells lack a perceptible adherence to mucus (Figure 6). As inferred by this result, the SpaF pilin subunit would then seem to be the main mucus-specific binding determinant of the recombinant-assembled SpaFED pilus.

**Figure 7:**
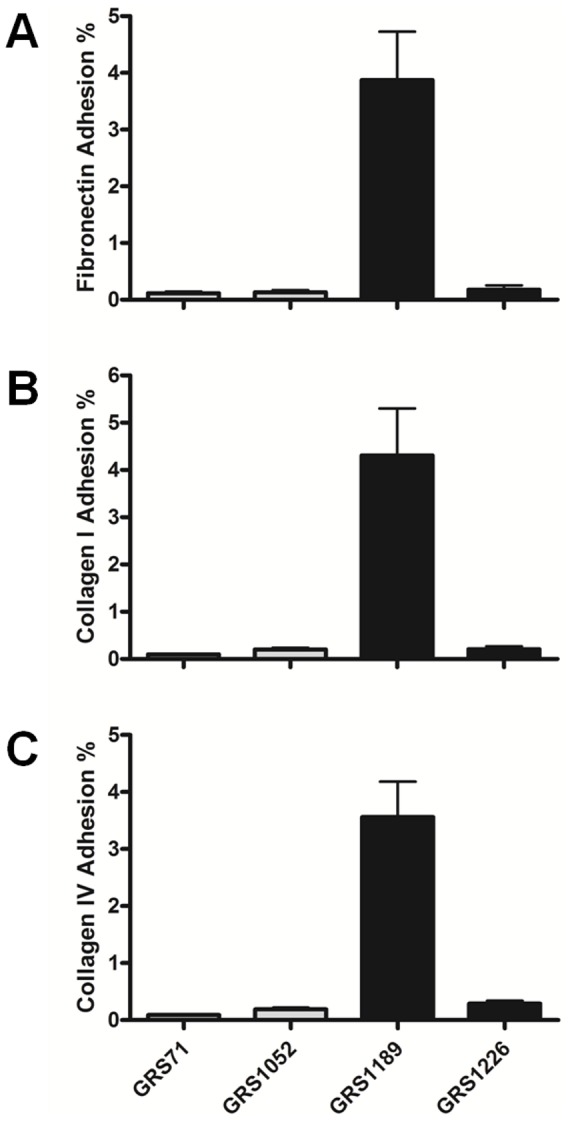
Binding of recombinant SpaFED-piliated lactococcal cells to ECM proteins. *In vitro* binding specificities between the normalized (OD600 = 0.5) cultures of recombinant WT (GRS1189) and SpaF pilin-deleted (GRS1126) SpaFED-piliated lactococci (along with GRS71 and GRS1052 cells as controls) and the fibronectin (**A**), collagen I (**B**), and collagen IV (**C**) proteins were evaluated using the procedure described in Materials and Methods. Triplicate measurements were made for each of the ECM protein experiments, each of which was performed independently twice. SEM is indicated as error bars. For all ECM proteins tested, differences between the GRS1189 and GRS1226 binding results from pairwise comparisons against the GRS71 data are regarded very significant (*P*≤0.005).

In some of our earlier work (unpublished data), we had observed that the recombinant-produced SpaF and SpaC pilins did not bind detectably to a representative panel of extracellular matrix (ECM) proteins, although subsequently it was later revealed, and in this case by more sophisticated means [7], that the SpaC subunit shows some measurable adherence to collagen. With this finding in mind, we decided to re-examine the *in vitro* binding specificity of SpaF by testing the adhesion ability of SpaFED-piliated GRS1189 and GRS1226 lactococcal cells toward certain ECM proteins. Here, and as indicated in Figures 7A-C, it is more than apparent that nisin-induced GRS1189 cells show considerable binding to fibronectin protein, and as well to both the collagen I and IV proteins. Moreover, compared to the controls used (i.e., the GRS71 and GRS1052 cells), and as anticipated, the measured adhesion levels are much more pronounced and significant with the WT SpaFED-piliated GRS1189 lactococci. More importantly though, this specific binding capacity, which we infer is due to the surface-localized SpaFED pili, could be credited to the SpaF pilin, as the GRS1226 cells, whose recombinant surface piliation is missing the SpaF subunit, did not any longer adhere to these individual ECM proteins (Figures 7A-C).

**Figure 8:**
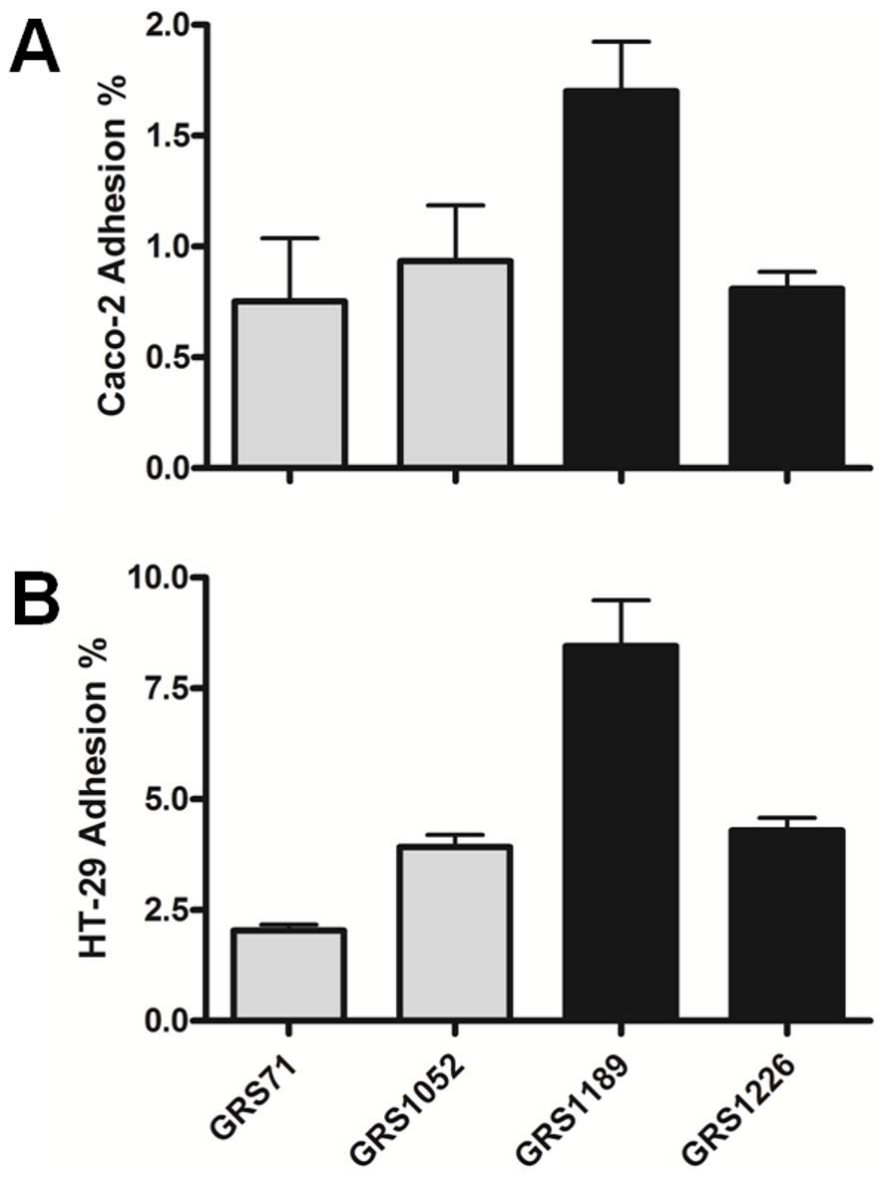
Adhesive interactions between recombinant SpaFED-piliated lactococci and human intestinal cell lines. Cell-to-cell adhesion assays involving the Caco-2 (**A**) and HT-29 (**B**) gut-related epithelial cell lines and the normalized (OD600 = 0.5) cultures of recombinant WT (GRS1189) and SpaF pilin-deleted (GRS1126) SpaFED-piliated lactococci, including GRS71 and GRS1052 cells as controls, were performed as outlined in Materials and Methods. Triplicate measurements were taken for both sets of experiments. Caco-2 and HT-29 binding experiments were carried out independently four and three times, respectively. SEM is displayed as error bars. For both of the intestinal cell lines, pairwise comparisons made between GRS1189 or GRS1226 cells and the GRS71 control strain indicate the differences in binding data are statistically extremely significant (*P*≤0.0001).

As a way to gauge whether the substrate binding specificities so described above for recombinant-produced SpaFED pili would also be relevant for mediating cell-to-cell interactions, we decided to analyze further the binding properties of the recombinant SpaFED-piliated GRS1189 and GRS1226 lactococci by evaluating their abilities for adherence to two different gut-related epithelial cell lines, so-called Caco-2 and HT-29. As clearly shown by *in vitro* adhesion assays, significant binding between WT-piliated GRS1189 lactococci and either Caco-2 or HT-29 cells is quite apparent and readily detected (Figures 8A and B), and as well, with the corresponding measured values more than exceeding those for the SpaFED pilus-less lactococcal cells being used as controls (i.e., GRS71 and GRS1052). Also, as would be expected, the relative adhesion levels between GRS1226 lactococci and the two intestinal cell lines appear to be markedly reduced (Figures 8A and B). Once again and based on these results, one can then surmise that the SpaF subunit is wielding a focal adhesiveness and thus most likely is the responsible determinant for SpaFED pilus-mediated binding with Caco-2 and HT-29 cells, and as well if one speculates with specific applicability in mind, perhaps with those epithelial cells that actually form the mammalian gut lining.

Irrespective of the fact that in *L. rhamnosus* GG the occurrence of SpaFED piliation is still considered a hypothetical externally-expressed structural feature, for interpreting the above binding experiments in a natural context the actual cellular production of these pili might nonetheless be envisaged and speculated upon as such. Here then, with its combined and targeted binding functionalities to intestinal substrates (i.e., mucus, ECM proteins, and gut epithelial cells), the SpaFED pilus would then provide the *L. rhamnosus* GG strain with an even more augmented and finely tuned capacity for residing within the intestinal tract, as already being offered by other mucoadhesive surface structures [4], [28], [29]. This is yet more evident when the epithelial intestinal lining is breached by injury or disease, as this would cause the different underlying ECM proteins to become exposed and unprotected, thereby rendering them easily accessible to various gut-dwelling bacteria, like *L. rhamnosus* GG itself. In a more widely applicable stance as a probiotic commensal bacterium, *L. rhamnosus* GG would be better able to convey its perceived host-cell benefits for what might then be judged as a conceivably even longer duration. However, since the types and identities of stimuli (if any) that would in effect trigger SpaFED pilus production in *L. rhamnosus* GG cells is still a conundrum to be solved, it should be reiterated that any proposed *in vivo* function for SpaFED piliation during host-cell adhesion processes must be accompanied with ample circumspection.

### Molecular immunogenicity of lactococcal-expressed SpaFED pili

Rather recently, we carried out a study whose aim was to further an understanding of what possible role might be played by the SpaCBA pilus during *L. rhamnosus* GG cell interactions with the intestinal immune system [9]. For this, we had used *L. lactis* cells expressing recombinant-assembled SpaCBA pili, and while serving as a useful molecular tool they helped show that the SpaCBA pilus might participate in gut-immune crosstalk. Here, as implicative evidence, we observed that this surface appendage was able to mediate Toll-like receptor (TLR) signaling and inflammatory cytokine production activities in immune-related cells [9]. From these results, we proposed that the SpaCBA pilus can be considered a new type of microbe-associated molecular pattern (MAMP)-like modulator of innate immunity, and also that it is now one of several other cell-surface structures exerting an immunomodulating function in *L. rhamnosus* GG [9]. Rather significantly, we also concluded that the ancillary SpaC pilin with its adhesiveness is a determining factor for SpaCBA pilus-induced immuno-responsiveness. Having then established this level of immune functioning for SpaCBA piliation, we decided to use the GRS1189 and GRS1226 lactococcal clones and perform a related set of immunological experiments to examine whether or not recombinant SpaFED pili might possess an analogous type of molecular immunogenicity.

**Figure 9:**
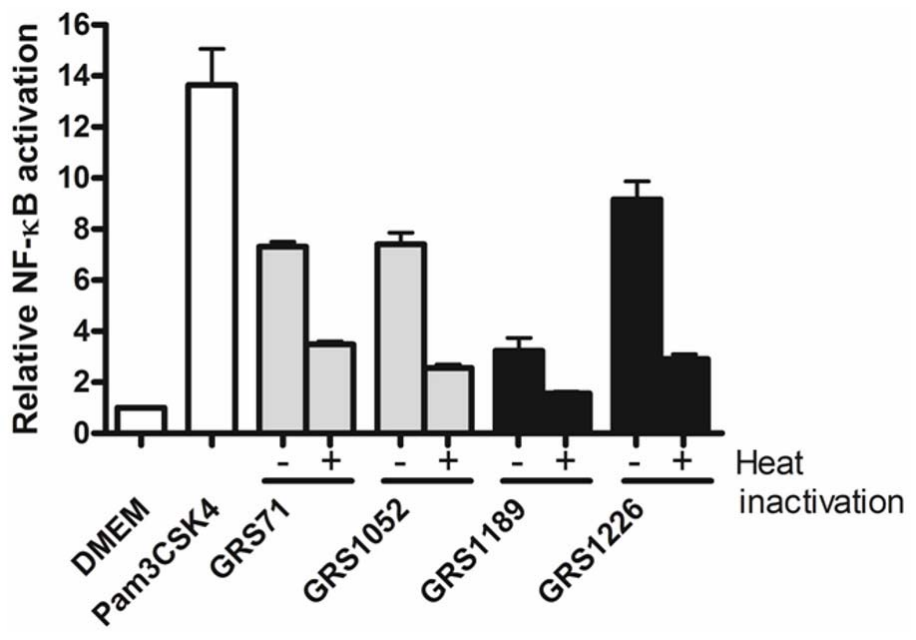
Stimulation of TLR2-dependent NF-κB activation by recombinant SpaFED-piliated lactococci. The HEK-TLR2 cell line was treated with live (−) or heat-treated (100°C for 10 minutes) (+) normalized cultures of recombinant WT (GRS1189) and SpaF pilin-deleted (GRS1126) SpaFED-piliated lactococci (MOI 100). Levels of TLR2-dependent NF-κB activation were assessed as described in Materials and Methods. Testing of the GRS71 and GRS1052 control strains was as well conducted. DMEM cell-culture medium and a TLR2-agonist lipopeptide (Pam3CSK4; 1 ng/ml) served as negative and positive controls, respectively. Quadruplicate measurements were taken for two independent experiments. SEM is shown as error bars. Pairwise differences between the GRS1189 and GRS1226 data (without heat inactivation) are considered very significant (*P*≤0.005).

For the first immuno-characterizations, we investigated whether the SpaFED pilus can act as a TLR2 agonist, with this then being done by following the same strategy we had used when studying SpaCBA surface piliation [9]. Briefly, SpaFED-piliated GRS1189 and GRS1226 lactococcal cells (post-nisin induction) were tested for their ability to induce a HEK293 cell line carrying the gene for human TLR2 and those for a NF-κB-regulated secreted alkaline phosphatase (SEAP) reporter system, with the latter used to monitor the extent of TLR2 signaling. However, as indicated in Figure 9, and somewhat unexpected in the context of our recent findings with SpaCBA pili [9], GRS1189 cells with WT SpaFED piliation are unable to stimulate TLR2-dependent activity at even the same levels as detected with the GRS71 and GRS1052 cells. These latter cells were intended for use as negative controls, but they themselves on their own seem to elicit measurable TLR2 responses in the HEK293 cells, and as well, much more so than SpaFED-piliated GRS1189 lactococci. In contrast, however, with the elimination of the SpaF pilin from the SpaFED pilus structure in GRS1226 lactococci, these cells regained their potency, as it now appears the induction of NF-κB activation in HEK-TLR2 cells is equivalent to the levels being detected with the GRS71 and GRS1052 cells (Figure 9). We interpret these results to mean that the SpaFED pilus is itself acting to dampen lactococcal cell-induced TLR2-related signaling (as seen with the GRS71 and GRS1052 cells), and key to this apparent outcome is the SpaF pilin, which speculatively might possibly involve its inherent adhesive nature. Surprisingly, this is exactly the opposite effect we found with recombinant SpaCBA pili [9], and, to some extent, that which was reported for bifidobacterial [30] and streptococcal [31] sortase-dependent pili.

**Figure 10:**
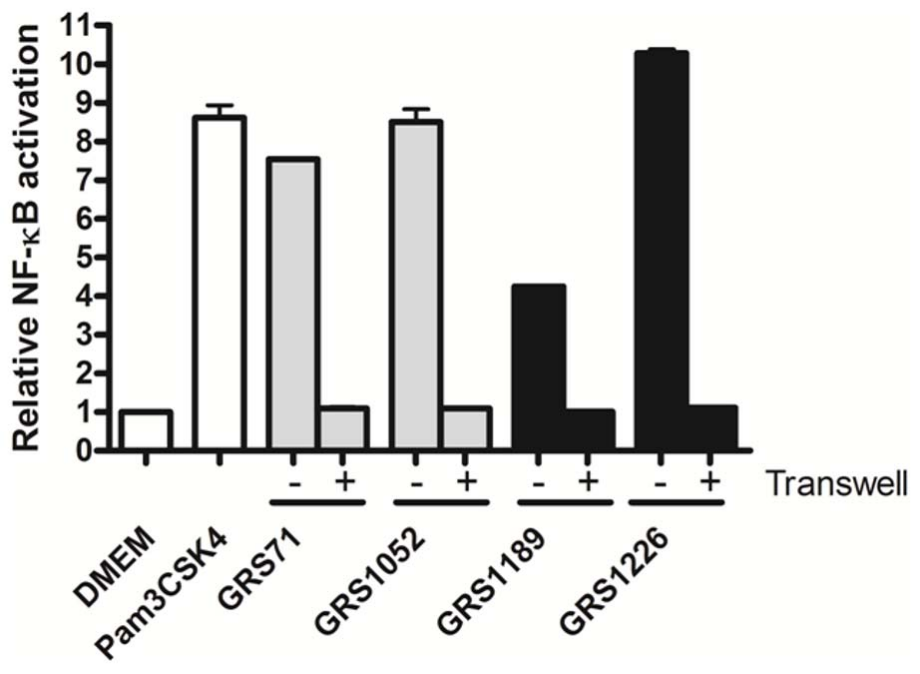
Effect of cell-to-cell contacts on TLR2-induced NF-κB activation by recombinant SpaFED-piliated lactococci. Using a Transwell membrane-segregated system, HEK-TLR2 cells were treated with non-partitioned (−) and partitioned (+) normalized cultures of recombinant WT (GRS1189) and SpaF pilin-deleted (GRS1126) SpaFED-piliated lactococci (MOI 100). Monitoring of TLR2-dependent NF-κB activation was carried out as outlined in Materials and Methods. Included as controls were GRS71 and GRS1052 cells (MOI 100), DMEM cell-culture medium, and Pam3CSK4 (1 ng/ml). Triplicate measurements were taken for a single experiment. SEM is indicated as error bars.

Done in parallel to corroborate whether the TLR2-dependent activation being augmented is due to a proteinaceous entity, we also performed these experiments using heat-treated living lactococcal cells, whereupon afterward we analyzed the spent culture supernatant for TLR2-activated NF-kB signaling responses (Figure 9). Of most relevance here, we found that with the SpaFED-piliated GRS1189 and GRS1226 cells, and following their exposure to a temperature that was enough to cause denatured protein folding, this then all but eliminates NF-κB activation in HEK-TLR2 cells (Figure 9). Inferred from this, it is reasonable to assume that heat-labile proteins are being affected, thus suggestively implicating, among other lactococcal-associated proteins, the involvement of SpaFED pili. In addition, to verify that the effects we observed were from proteins that are held closely and firmly to the cell surface, experiments were performed using Transwell cell culture membranes, which then would prevent any cell-to-cell contact between the GRS1189 and GRS1226 lactococci and the HEK-TLR2 cells. As inferred from Figure 10, TLR2 actions being measured are not due to cell-released protein, but rather depend on surface proteins that presumably remain fixed through attachment to the cell-wall structure.

**Figure 11:**
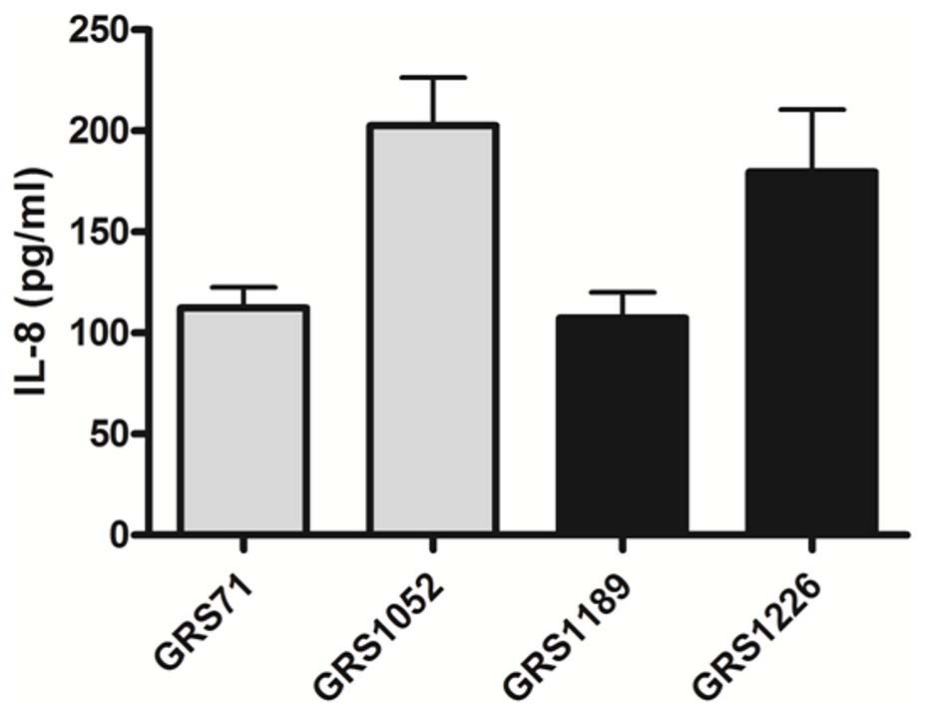
Induction of IL-8 cytokine production in Caco-2 cells by recombinant SpaFED-piliated lactococci. Caco-2 cells were treated with normalized cultures of recombinant WT (GRS1189) and SpaF pilin-deleted (GRS1126) SpaFED-piliated lactococci (MOI 100). GRS71 and GRS1052 cells (MOI 100) were used as controls. Endogenous IL-8 cytokine production levels in spent cell culture supernatants were measured as described in Materials and Methods. Triplicate measurements were taken for the experiments, which were repeated independently four times. SEM is displayed as error bars. Pairwise differences between GRS1189 and GRS1226 data are deemed significant (*P*≤0.05).

**Figure 12:**
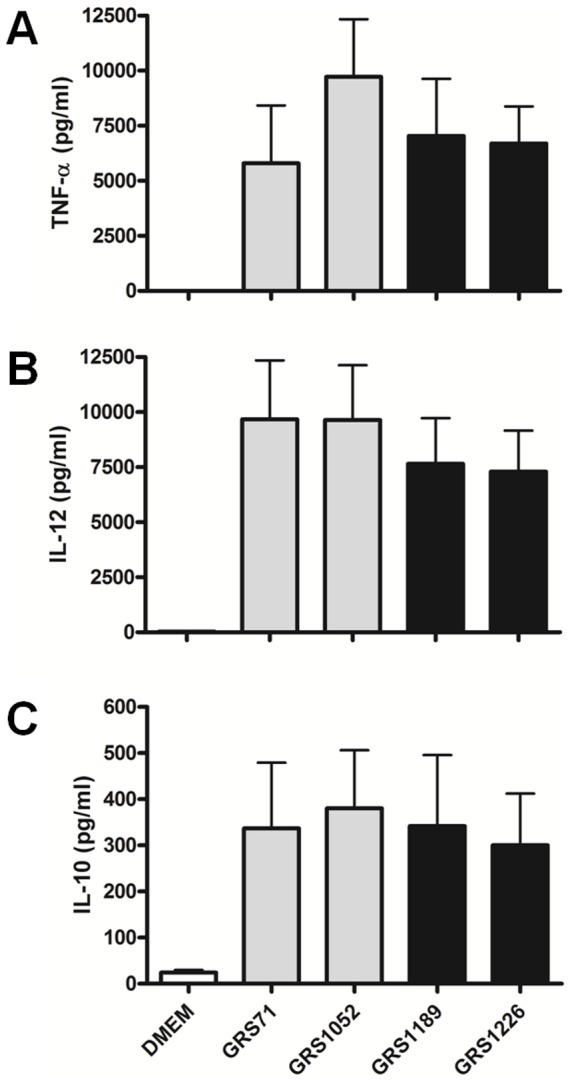
Stimulation of DC-cytokine production by recombinant SpaFED-piliated lactococci. Human monocyte-derived dendritic cells (moDCs) were treated with normalized cultures of recombinant WT (GRS1189) and SpaF pilin-deleted (GRS1126) SpaFED-piliated lactococci (MOI 50). Stimulated TNF-α (**A**), IL-12 (**B**), and IL-10 (**C**) cytokine production was measured as described in Materials and Methods. GRS71 and GRS1052 cells (MOI 50) and DMEM cell-culture medium were included as controls. Measurements were performed in triplicate using moDCs from four new and different donors each time. SEM is shown as error bars. For all tested cytokines, differences in a pairwise comparison between the GRS1189 and GRS1226 data are judged not significant (*P*≥0.05).

For our other immuno-characterization experiments, we tested whether the SpaFED pilus-driven dampening effect is distinctive and extends to other immune responses in different host-immune cells. Here, we measured what changes occurred to the endogenous interleukin-8 (IL-8) levels in Caco-2 intestinal cells after being treated with the SpaFED-piliated GRS1189 and GRS1226 lactococcal clones, and as well for comparisons with the GRS71 and GRS1052 “control” strains. As such, it can be seen from Figure 11 that SpaFED pilus-associated dampening is also noticeable in this gut epithelial cell line, as the corresponding IL-8 levels are lowered with GRS1189 cells, but in fact do make a recovery with the GRS1226 cells, wherein the pili being expressed no longer consist of the SpaF pilin subunit. Contrastingly, however, when an analogous experiment was repeated using human monocyte-derived dendritic cells (moDCs), and where the fluctuations in the levels of tumor necrosis factor-alpha (TNF-α) and interleukins IL-12 and IL-10 were then monitored, no appreciable dampening in the production of these pro- and anti-inflammatory cytokines could be discerned with the SpaFED-piliated GRS1189 cells (Figures 12A-C). In fact, nor is it that the recombinant-produced SpaFED pili can potentiate lactococcal cell-induced DC-cytokine production levels like what was described previously for SpaCBA piliation [9]. While this particular result is perplexing in itself, we suspect that with the derived source of the moDCs being blood this then might have an impact on the nature of any bacteria-induced host-immune responses, as these could be construed as less reflective of intestinal cells within the context of the gut micro-environment.

The aforementioned immuno-characterizations certainly do highlight a striking functional difference in the molecular immunogenicity between *L. rhamnosus* GG *spaFED*- and *spaCBA*-encoded surface pili. Supposedly then, it can be said that for the so-hypothesized SpaFED pilus in *L. rhamnosus* GG, but here if it was actually natively expressed, such a surface appendage would likely offer some other targeted (and unique) immunogenic function for this gut-adapted bacterium. Moreover, one might go on to speculate further that any intestine-dwelling lactobacilli having simultaneously endogenous-expressed forms of SpaCBA and SpaFED piliation are then conceivably able to coordinate an induced-harmonizing counterbalance between the raised and lowered immuno-responsiveness of host cells, thereby helping to sustain a localized homeostatic aspect of immune tolerance in the mammalian gut surroundings.

## Concluding Comments

Given the provisional and conjectural nature of an “expressed” SpaFED pilus in *L. rhamnosus* GG (but as well in certain other strains and among related *Lactobacillus* species), it is pertinent to emphasize that for the aforesaid results, and apart from the corresponding data having been assessed and interpreted with the proper biological context in mind, our broad inferences regarding this gut-commensalic bacterium should nonetheless be treated with some level of circumspect. However, as an attempt to shift the existing view of the *spaFED* operon-encoded pilus from a hypothetical premise to one that is physically more tangible or observable, we had confined our efforts to revealing the gross structural arrangement of its recombinant synthetic form, and as well to evaluating how this so-assembled structure then behaves in terms of functional action and specificity. In this regard, our present study has yielded some interesting and relevant results, but more pointedly it has provided the first phenotypic glimpse of the so-perceived dormant fimbrial *spaFED* operon. Key findings we consider most significant are highlighted briefly as follows:

- The *L. rhamnosus* GG fimbrial *spaFED* operon is itself a functioning translational unit when cloned in *L. lactis*, whereupon it can encode the production of a prototypical sortase-dependent pilus, which not only is adept at cell-surface attachment, but as well, is having a multi-subunited structure comprised of the tip-positioned SpaF, basal SpaE, and backbone SpaD pilin-proteins.
- In its assembled and anchored form, and via recombinant expression in lactococci, the *L. rhamnosus* GG SpaFED pilus displays a multi-targeted binding specificity, as clearly evidenced by its ability to promote *in vitro* cellular adhesion to mucus components, collagen (I and IV) and fibronectin proteins, and intestinal-type epithelial cells (Caco-2 and HT-29). For these pilus-mediated binding functionalities, the SpaF pilin subunit is seen as the main adhesive determinant.
- In stark difference to the immunopotentiating effect reported for SpaCBA piliation [9], recombinant-produced *L. rhamnosus* GG SpaFED pili are unable to “boost” some of the host-innate immune responses being endogenously elicited by lactococcal cells. Rather on the contrary, this particular pilus form behaves reversely to a so-anticipated precedent and instead serves to dampen or minimize the immunogenic-related reactions being triggered in certain types of intestinal- and immune-related cells.

## Materials and Methods

### Bacterial strains, growth media, and cultivation conditions

*Lactococcus lactis* NZ9000 (*pepN::nisRnisK*) [32] (designated as GRS71) was originally derived from the *L. lactis* MG1363 strain and served as the cloning host for recombinant production of *L. rhamnosus* GG SpaFED pili. Typically, cells were cultivated overnight with M17 medium (Difco) and 0.5% glucose (GM17) either on agar plates or in static liquid broths at 30°C. The growth of recombinant pilus-producing lactococci requires GM17 media to be supplemented with 7.5 µg/ml chloramphenicol. *Lactobacillus rhamnosus* GG (ATCC 53103) was as a rule grown overnight at 37°C using either solid or liquid MRS (de Man-Rogosa-Sharpe) media (Difco).

### Cell lines and culturing conditions

The HEK-Blue-hTLR2 cell line was obtained commercially from InvivoGen and represents HEK293 cells with the genes for human TLR2 and a NF-κB-inducible SEAP (secreted embryonic alkaline phosphatase) reporter system, which itself allows for the monitoring of TLR2 signaling. The culture medium routinely used was Dulbecco's modified Eagle's medium (DMEM) with 4.5 g/l glucose, and as well contained 10% (v/v) heat-inactivated fetal calf serum (FCS), 2 mM L-glutamine, 100 µg/ml Normocin, 50 U/ml penicillin, and 50 µg/ml streptomycin. When it was required, this modified DMEM medium was also supplemented with HEK-Blue selection antibiotics. HEK293 cells were typically cultured using the manufacturer-specified cell media conditions and as was essentially described in our previous work [9]. The Caco-2 cell line was from our in-house cell culture collection, and the corresponding cells were ordinarily grown in Roswell Park Memorial Institute (RPMI) 1640 medium containing 25 mM HEPES buffer and 2.2 mg/ml NaHCO_3_, which was then supplemented with 10% FCS, 2 mM L-glutamine, 50 µg/ml gentamicin, and 1% nonessential amino acids. The HT-29 cell line was purchased from the culture collection of Public Health England (PHE) (Department of Health, United Kingdom), with the cells grown and maintained in McCoy's 5A modified medium that contains 10% FCS, 2 mM L-glutamine, 50 U/ml penicillin, and 50 µg/ml streptomycin. Caco-2 and HT-29 cells are originally derived from a human colorectal adenocarcinoma, both exhibiting typical epithelial morphology, but with the HT-29 cell line able to secrete more mucus once becoming fully differentiated. Cell lines were normally incubated for three (Caco-2) or two (HT-29) weeks at 37°C in a humidified 5% CO_2_ atmosphere until 90% confluence, at which point they are then used for seeding out experiments. Unless otherwise mentioned, all culture media and other supplemented ingredients were purchased from Gibco or Sigma.

### DNA plasmids and recombinant manipulations

*Lactococcus* cloning was performed using the pKTH5080 plasmid (unpublished), which itself was constructed from a lactococcal expression vector (pNZ8032 [33]) that had the regulatory genes (*nisR* and *nisK*) and promoter site (P*nisA*) for nisin-inducible gene expression. Added extra to create pKTH5080 were the DNA sequences for the S-layer protein (SlpA) secretion signal and transcriptional terminator regions in *Lactobacillus brevis*, and as well, for new *Eco*RI and *Xho*I restriction endonucleases in the multiple cloning site. Plasmid DNA from *L. lactis*, and as well genomic DNA from *L. rhamnosus* GG, were both isolated and recovered using commercially available kits and done with some minor modifications to the recommended protocols of the manufacturer. For molecular cloning practices (e.g., PCR amplification, restriction endonuclease digestion, and DNA ligation), conventional DNA approaches and techniques were employed.

### PCR cloning of plasmid constructs for SpaFED pilus expression in *L. lactis*

Lactococcal expression plasmids for producing WT and SpaF pilin-deleted *L. rhamnosus* GG SpaFED pili were made using established PCR cloning techniques. Here, with chromosomal DNA from *L. rhamnosus* GG serving as the template, single-length fragments of DNA encompassing the genes of the fimbrial *spaFED* operon, either *spaF*, *spaE*, *spaD*, and *srtC2* for cloning the WT construct or *spaE*, *spaD*, and *srtC2* for cloning the SpaF pilin-deleted one, were each amplified by PCR using a pair of specific oligonucleotide primers that as well introduced the sequences for *Nco*I and *Xho*I restriction sites at the 5′- and 3′-ends, respectively. For this, the *Nco*I-containing forward primers, 5′-AGTGAAAAATGTA*CCATGG*AAGGAGGCAC (WT) and 5′-GAGGCCCGTTA*CCATGG*GACGATTTTATTG (Δ*spaF*) [*Nco*I site is italicized], and the *Xho*I-containing reverse primer, 5′-TCTTACTTTCTAACATT*CTCGAG*CCAGATTACG (WT and Δ*spaF*) [*Xho*I site is italicized], were used (Oligomer Oy, Finland). Once both amplified PCR fragments were recovered from agarose gels, they were treated with *Nco*I and *Xho*I restriction endonucleases, and following each of their ligations into the pKTH5080 nisin-inducible expression plasmid, these then were electroporated into competent *L. lactis* NZ9000 (GRS71) cells using a protocol described previously [34]. Recombinant lactococcal transformants were identified by their antibiotic resistance on chloramphenicol-supplemented (7.5 µg/ml) solid GM17 growth medium. Following confirmation by PCR screening, transformant clones having plasmids with the right-sized *spaFED* operon insert were selected for further use. Lactococcal clones with the WT (pKTH5393) and SpaF pilin-deleted (pKTH5443) plasmid constructs were called GRS1189 and GRS1226, respectively. *L. lactis* GRS1052 (unpublished), a lactococcal clone carrying the non-inserted pKTH5080 plasmid, was used as a control.

### Nisin-induced production of recombinant SpaFED pili in *L. lactis*

Cultivation starters of the recombinant GRS1052, GRS1189, and GRS1226 lactococcal clones were obtained by growing cells overnight at 30°C in a static chloramphenicol-supplemented (7.5 µg/ml) GM17 liquid broth. After a 1∶25 dilution of these cells with the same growth medium, they were cultivated at 30°C until the optical density at 600 nm (OD600) reached near 0.4 to 0.5, whereupon *spaFED*-encoded pilus production was initiated with nisin. For this, nisin was sourced from the nisin-overproducing *L. lactis* NZ9700 strain [35] (provided as a gift by Dr. François Douillard, University of Helsinki) and added as a sterile-filtered cell-free supernatant (0.2%, v/v) that had been made from an overnight grown culture. Once having been nisin-induced, pilus production and growth of the recombinant lactococci was continued overnight at 30°C, after which the cells were centrifugally recovered, rinsed once with phosphate-buffered saline (PBS) (pH 7.2), and depending on their planned use then resuspended in cell culture medium, PBS, or SDS-PAGE loading buffer.

### Immunoblotting detection

Overnight-grown recombinant lactococci were pelleted, with the cells then being rinsed with PBS and resuspended in a small volume of 10 mM Tris-HCl (pH 6.8) buffer. This cell suspension was mixed with an equal volume of 3× gel loading buffer (135 mM Tris-HCl, pH 6.8, 30% glycerol, 3% SDS, and 0.03% bromophenol blue), sonicated for a short time, heated to 100°C for at least 5 minutes, and then centrifuged briefly. Small-sized aliquots were then taken from the supernatant and the protein content resolved by conventional SDS-PAGE using precast 4–20% gradient gels (Bio-Rad). These gels were electroblotted onto Immobilon-P (polyvinylidene difluoride) membranes (Millipore) and the corresponding *L. rhamnosus* GG pilin-proteins (SpaF, SpaE, and SpaD) then detected with each of their respective antisera, which were produced previously in rabbits and directed against recombinant-produced protein [6]. Membranes were subsequently probed with a secondary antibody (horseradish peroxidase-conjugated goat anti-rabbit IgG; Bio-Rad), and following this, the various pilus proteins were visualized by chemiluminescent means using the Amersham ECL Advance Western Blotting Detection Kit (GE Healthcare) and done according to the conditions recommended in the accompanying instructions.

### Immunogold transmission electron microscopic analysis

The method used for the immunogold pilin-labeling and electron microscopy (EM) of SpaFED-piliated lactococcal cells was adapted from the approach used by Ref. [36], and essentially done as had been described therein, albeit with some minor procedural changes. Both single- and double-labeling EM experiments were performed in the present study, and for this formvar-carbon-coated copper grids were used, along with rabbit antiserum against recombinant-produced SpaF, SpaE, and SpaD pilin-protein (diluted 1∶25 to 1∶100) [6] as the primary antibody and protein A conjugated to 10- and 15-nm diameter gold particles (diluted 1∶20) as the secondary antibody. Grids with piliated lactococcal cells were negatively stained with a methylcellulose-uranyl acetate solution. Highly magnified images were obtained by means of a JEOL JEM-1400 transmission electron microscope available at the Electron Microscopy Unit (Institute of Biotechnology) at the University of Helsinki.

### Mucus adhesion assay

The binding ability of nisin-induced recombinant lactococci to mucus was determined using an earlier described microtiter plate assay method [4], [6]. For this, tritiated thymidine metabolic labeling of *Lactococcus* cells was performed and the cell numbers then adjusted to an OD600 of 0.5 with PBS buffer. Radiolabeled cells (100 µl per well) were added to 96-well microtiter plates (Corning) that are precoated overnight with 50 µg mucus (porcine mucus type II; Sigma), and thereafter allowed to incubate at room temperature for 2 hours. Following careful rinsing (three times) with PBS to facilitate removal of any lightly mucus-attached cells, those wells with bacteria still bound to the immobilized mucus were treated with a 600-µl aliquot of lysis solution (1% SDS-0.1 N NaOH) and then incubated overnight at 37°C. Lysed cell suspensions were mixed with a 1-ml volume of OptiPhase “HiSafe” III scintillation liquid (Perkin-Elmer Life Sciences), and the radioactive counts were then measured using a liquid-scintillator detector. Mucus adhesion (as a percent) represents the measured amounts of radioactivity in the lysed cell suspension relative to those in the cell suspension added initially to the wells.

### Collagen and fibronectin adhesion assay

Assessing the ability of nisin-induced recombinant lactococci to bind ECM proteins was done using essentially the same procedure described above to measure cellular adhesion to mucus [4], [6]. Here, however, the 96-well microtiter plates were subjected to an overnight (refrigerated) precoating with a 1-µg amount of collagen (types I and IV) or fibronectin protein (Sigma). After this, but prior to adding the radiolabeled cells, each of the ECM protein-coated wells was pretreated with a blocking solution (5% skimmed milk) for 2 hours at room temperature. The amount of collagen or fibronectin binding activity was calculated as a percentage of the detected radioactivity, just as is done when quantifying mucus adhesion (see above).

### Intestinal epithelial cell line adhesion assay

The assay method for measuring adherence between nisin-induced recombinant lactococci and two different intestinal epithelial cell lines was based on the protocol used for determining bacterial adhesion to mucus and ECM proteins (see previous sections). For this, however, there were some procedural modifications requiring to be implemented. For instance, confluent (∼90%) Caco-2 and HT-29 cells were prepared as described already (see above) and used to seed 24-well cell culture plates (Corning) at about 1.0×10^4^ cells per well. Cell lines were grown for three (Caco-2) or two (HT-29) weeks at 37°C with 5% CO_2_, with each of the epithelial cell-containing wells then being rinsed twice with FCS-free culture media, to which afterward were added 600-µl volumes of radiolabeled recombinant lactococci that had been normalized to OD600 = 0.5 with the same cell media. This mixed suspension of bacterial and intestinal epithelial cells was then allowed to incubate for about 2 hours in the presence of 5% CO_2_ and while at 37°C. The remaining part of the adhesion assay is continued as described above for the mucus- and ECM protein-binding determinations, and in the same way the percent of cell adhesion is calculated from the radioactivity data.

### Induced activation of human HEK-TLR2 cells

Experiments for the recombinant lactococcal cell-induced activation of human HEK-TLR2 cells were conducted using essentially the same methods as described in our previous study [9]. Briefly, 24-well culture plates were seeded with the HEK-TLR2 cell line at around 5.0×10^4^ cells per well and grown overnight in culture media without selection antibiotics (see above). These cells were treated with recombinant lactococci (or otherwise indicated) using a multiplicity of infection (MOI) of 100 and the levels of TLR2-induced NF-kB activation in culture supernatants then underwent measurement on the next day. For this, a 20-µl aliquot of the culture supernatant was mixed together with a 180-µl volume of pre-warmed QUANTI-Blue reagent in a 96-well microtiter plate, which was then incubated at 37°C. SEAP production was then quantified by spectrophotometric means at 620 nm using QUANTI-Blue detection medium as specified in the manufacturer-recommended instructions, with the extent of color development being assessed at various time points (i.e., 15, 60, 120, and 180 minutes). Triplicate measurements were typically taken, unless noted otherwise. Additional HEK-TLR2 cell-related experiments involving heat-treatments and the use of Transwell cell culture membrane inserts (Becton Dickinson & Company; 0.4-µm pore size) were both carried out as done in our earlier work [9].

### Induced endogenous cytokine production in the human Caco-2 cell line

Caco-2 cells were cultivated and prepared as described above. Recombinant lactococcal cell-induced deviations to the endogenous IL-8 cytokine content in this cell line were demonstrated by measuring cell culture supernatants using the BD OptEIA™ ELISA kit and according to the instructions therein provided by the manufacturer (BD Biosciences).

### Induced endogenous cytokine production in the human dendritic cells

Human monocyte-derived dendritic cells (moDCs) were targeted for measuring recombinant lactococcal cell-stimulated fluctuations to the cytokine production profile in primary immune cells. The approach used for isolating and generating moDCs was according to the same protocols and methods that had been described in our earlier published study [9]. Recombinant lactococcal cells were grown overnight and prepared as already mentioned above, and then thereafter their optical densities (OD600) were normalized with a RPMI 1640 medium that also included 10% FCS, antibiotics, L-glutamine, and HEPES, but which was free of interleukin-4 (IL-4) and granulocyte-macrophage colony-stimulating factor (GM-CSF). Typically, moDCs were exposed to bacteria using a MOI of 50, and then for approximately 24 hours they were allowed to incubate at 37°C with 5% CO_2_. After this, the cell culture supernatants were obtained and then at this point the variability in the cytokine (i.e., TNF-α, IL-10, and IL-12) levels was measured by ELISA using the BD OptEIA™ ELISA kit (BD Biosciences). Bacteria-treated moDCs were from four different donor samples, with each of their equivalent cell culture supernatants being recovered and then examined separately. These experiments were normally performed in triplicate.

### Statistical analysis

The statistical relevance of the accompanying experimental data from this study was estimated by using the GraphPad Prism statistical software package (version 4.0). For this, pairwise comparisons and correlations were done using the unpaired Student's *t* test, with the calculated *P* values being assigned as significant (0.05 or less), very significant (0.005 or less), extremely significant (0.001 or less), or not significant (0.05 or more).

## Supporting Information

Figure S1 **Multiple sequence alignment of nucleotides encompassing the fimbrial** ***spaFED*** **-operon promoter region.** A multiple alignment of a ∼600-nucleotide (nt) length of sequence immediately upstream of the *spaF* locus of the fimbrial *spaFED* operon is shown. Nucleotide sequences encompassing this region were recovered from the genomes of the following *L. rhamnosus* strains: GG, ATCC 53103, PEL6, PEL5, LRHMDP2, LRHMDP3, LMS2-1, ATCC 8530, LC705, HN001, E800, R0011, and ATCC 21052. Nucleotide sequences were aligned using the MultAlin program [37] (http://multalin.toulouse.inra.fr/multalin/multalin.html). Nucleotides matching exactly the consensus sequence and found in all aligned sequences are marked in red. Nucleotides that deviate from the consensus sequence are marked in either blue (for the majority) or black (for the minority).
(PDF)
