# Supplementary material for: Phenotypical Analysis of the Lactobacillus rhamnosus GG Fimbrial spaFED Operon: Surface Expression and Functional Characterization of Recombinant SpaFED Pili in Lactococcus lactis
Source: PLoS One. 2014 Nov 21;9(11):e113922. doi: 10.1371/journal.pone.0113922 (PMC4240662; doi:10.1371/journal.pone.0113922)
Supplement: Figure S1 — Multiple sequence alignment of nucleotides encompassing the fimbrial spaFED -operon promoter region. A multiple alignment of a ∼600-nucleotide (nt) length of sequence immediately upstream of the spaF locus of the fimbrial spaFED operon is shown. Nucleotide sequences encompassing this region were recovered from the genomes of the following L. rhamnosus strains: GG, ATCC 53103, PEL6, PEL5, LRHMDP2, LRHMDP3, LMS2-1, ATCC 8530, LC705, HN001, E800, R0011, and ATCC 21052. Nucleotide sequences were aligned using the MultAlin program [37] (http://multalin.toulouse.inra.fr/multalin/multalin.html). Nucleotides matching exactly the consensus sequence and found in all aligned sequences are marked in red. Nucleotides that deviate from the consensus sequence are marked in either blue (for the majority) or black (for the minority). (PDF) [file pone.0113922.s001.pdf]

```
           1       10        20        30        40        50        60        70        80        90        100       110       120       130
GG         TTTCCGGTACAACGATGGTCCCGTTTGATGCCGAGCCTGCTAAAATGCACGTACCCAATGACAGCTATCCAGTAGCCGATGCTGACGGGCACTATGCTTATGACAAGCTCGTGGATTTAAAAGAGCTGCA
ATCC53103  TTTCCGGTACAACGATGGTCCCGTTTGATGCCGAGCCTGCTAAAATGCACGTACCCAATGACAGCTATCCAGTAGCCGATGCTGACGGGCACTATGCTTATGACAAGCTCGTGGATTTAAAAGAGCTGCA
PEL6       TTTCCGGTACAACGATGGTCCCGTTTGATGCCGAGCCTGCTAAAATGCACGTACCCAATGACAGCTATCCAGTAGCCGATGCTGACGGGCACTATGCTTATGACAAGCTCGTGGATTTAAAAGAGCTGCA
PEL5       TTTCCGGTACAACGATGGTCCCGTTTGATGCCGAGCCTGCTAAAATGCACGTACCCAATGACAGCTATCCAGTAGCCGATGCTGACGGGCACTATGCTTATGACAAGCTCGTGGATTTAAAAGAGCTGCA
LRHMDP2    TTTCCGGTACAACGATGGTCCCGTTTGATGCCGAGCCTGCTAAAATGCACGTACCCAATGACAGCTATCCAGTAGCCGATGCTGACGGGCACTATGCTTATGACAAGCTCGTGGATTTAAAAGAGCTGCA
LRHMDP3    TTTCCGGTACAACGATGGTCCCGTTTGATGCCGAGCCTGCTAAAATGCACGTACCCAATGACAGCTATCCAGTAGCCGATGCTGACGGGCACTATGCTTATGACAAGCTCGTGGATTTAAAAGAGCTGCA
LMS2-1     TTTCCGGTACAACGATGGTCCCGTTTGATGCCGAGCCTGCTAAAATGCACGTACCCAATGACAGCTATCCAGTAGCCGATGCTGACGGGCACTATGCTTATGACAAGCTCGTGGATTTAAAAGAGCTGCA
ATCC8530   TTTCCGGTACAACGATGGTCCCGTTTGATGCCGAGCCTGCTAAAATGCACGTACCCAATGACAGCTATCCAGTAGCCGATGCTGACGGGCACTATGCTTATGACAAGCTCGTGGATTTAAAAGAGCTGCA
LC705      TTTCCGGTACAACGATGGTCCCGTTTGATGCCGAGCCTGCTAAAATGCACGTACCCAATGACAGCTATCCAGTAGCCGATGCTGACGGGCACTATGCTTATGACAAGCTCGTGGATTTAAAAGAGCTGCA
HN001      TTTCCGGTACAACGATGGTCCCGTTTGATGCCGAGCCTGCTAAAATGCACGTACCCAATGACAGCTATCCAGTAGCCGATGCTGACGGGCACTATGCTTATGACAAGCTCGTGGATTTAAAAGAGCTGCA
E800       TTTCCGGTACAACGATGGTCCCGTTTGATGCCGAGCCTGCTAAAATGCACGTACCCAATGACAGCTATCCAGTAGCCGATGCTGACGGGCACTATGCTTATGACAAGCTCGTGGATTTAAAAGAGCTGCA
R0011      TTTCCGGTACAACGATGGTCCCGTTTGATGCCGAGCCTGCTAAAATGCACGTACCCAATGACAGCTATCCAGTAGCCGATGCTGACGGGCACTATGCTTATGACAAGCTTGTGGATTTAAAGGAGCTGCA
ATCC21052  TTTCCGGTACAACGATGGTCCCGTTTGATGCCGAGCCTGCTAAAATGCACGTACCCAATGACAGCTATCCAGTAGCCGATGCTGACGGGCACTATGCTTATGACAAGCTTGTGGATTTAAAGGAGCTGCA
Consensus  TTTCCGGTACAACGATGGTCCCGTTTGATGCCGAGCCTGCTAAAATGCACGTACCCAATGACAGCTATCCAGTAGCCGATGCTGACGGGCACTATGCTTATGACAAGCTcGTGGATTTAAAaGAGCTGCA

           131     140       150       160       170       180       190       200       210       220       230       240       250       260
GG         GAAGGCGTTCAAGGCAGCATTTGGTAGTGAAGATTTGCAATTAGATAAACGGGTTGTTTACATTCATGGTGTTCCGGACAGTTTGGAACTTCCAGATACGGTTAAAGGAACCGTCATGAACTATGATGCA
ATCC53103  GAAGGCGTTCAAGGCAGCATTTGGTAGTGAAGATTTGCAATTAGATAAACGGGTTGTTTACATTCATGGTGTTCCGGACAGTTTGGAACTTCCAGATACGGTTAAAGGAACCGTCATGAACTATGATGCA
PEL6       GAAGGCGTTCAAGGCAGCATTTGGTAGTGAAGATTTGCAATTAGATAAACGGGTTGTTTACATTCATGGTGTTCCGGACAGTTTGGAACTTCCAGATACGGTTAAAGGAACCGTCATGAACTATGATGCA
PEL5       GAAGGCGTTCAAGGCAGCATTTGGTAGTGAAGATTTGCAATTAGATAAACGGGTTGTTTACATTCATGGTGTTCCGGACAGTTTGGAACTTCCAGATACGGTTAAAGGAACCGTCATGAACTATGATGCA
LRHMDP2    GAAGGCGTTCAAGGCAGCATTTGGTAGTGAAGATTTGCAATTGGATAAACGGGTTGTTTACATTCATGGGGTTCCGGACAGTTTGGAACTTCCAGATACGGTTAAAGGAACCGTCATGAACTATGATGCA
LRHMDP3    GAAGGCGTTCAAGGCAGCATTTGGTAGTGAAGATTTGCAATTGGATAAACGGGTTGTTTACATTCATGGGGTTCCGGACAGTTTGGAACTTCCAGATACGGTTAAAGGAACCGTCATGAACTATGATGCA
LMS2-1     GAAGGCGTTCAAGGCAGCATTTGGTAGTGAAGATTTGCAATTGGATAAACGGGTTGTTTACATTCATGGGGTTCCGGACAGTTTGGAACTTCCAGATACGGTTAAAGGAACCGTCATGAACTATGATGCA
ATCC8530   GAAGGCGTTCAAGGCAGCATTTGGTAGTGAAGATTTGCAATTGGATAAACGGGTTGTTTACATTCATGGGGTTCCGGACAGTTTGGAACTTCCAGATACGGTTAAAGGAACCGTCATGAACTATGATGCA
LC705      GAAGGCGTTCAAGGCAGCATTTGGTAGTGAAGATTTGCAATTGGATAAACGGGTTGTTTACATTCATGGGGTTCCGGACAGTTTGGAACTTCCAGATACGGTTAAAGGAACCGTCATGAACTATGATGCA
HN001      GAAGGCGTTCAAGGCAGCATTTGGTAGTGAAGATTTGCAATTGGATAAACGGGTTGTTTACATTCATGGGGTTCCGGACAGTTTGGAACTTCCAGATACGGTTAAAGGAACCGTCATGAACTATGATGCA
E800       GAAGGCGTTCAAGGCAGCATTTGGTAGTGAAGATTTGCAATTGGATAAACGGGTTGTTTACATTCATGGGGTTCCGGACAGTTTGGAACTTCCAGATACGGTTAAAGGAACCGTCATGAACTATGATGCA
R0011      GAAGGCGTTCAAGGCAGCATTTGGTAGTGAAGATTTGCAATTAGATAAACGGGTTGTTTACATTCATGGCGTTCCGGACAGTTTGGAACTTCCAGATACGGTTAAAGGAACCGTCATGAACTATGATGCA
ATCC21052  GAAGGCGTTCAAGGCAGCATTTGGTAGTGAAGATTTGCAATTAGATAAACGGGTTGTTTACATTCATGGCGTTCCGGACAGTTTGGAACTTCCAGATACGGTTAAAGGAACCGTCATGAACTATGATGCA
Consensus  GAAGGCGTTCAAGGCAGCATTTGGTAGTGAAGATTTGCAATTgGATAAACGGGTTGTTTACATTCATGGgGTTCCGGACAGTTTGGAACTTCCAGATACGGTTAAAGGAACCGTCATGAACTATGATGCA

           261     270       280       290       300       310       320       330       340       350       360       370       380       390
GG         CACGTCACACTGCCAATTGCCGTGGGTAAAATTGTCCGCGCTTAGTAGCAAAATATAATTTAATAACGTAGGTTGTTCCCGATCCGTCTGAGCGTTTCCAGCTTAGTCAGGTCGGGATTTTTTGTGCGCC
ATCC53103  CACGTCACACTGCCAATTGCCGTGGGTAAAATTGTCCGCGCTTAGTAGCAAAATATAATTTAATAACGTAGGTTGTTCCCGATCCGTCTGAGCGTTTCCAGCTTAGTCAGGTCGGGATTTTTTGTGCGCC
PEL6       CACGTCACACTGCCAATTGCCGTGGGTAAAATTGTCCGCGCTTAGTAGCAAAATATAATTTAATAACGTAGGTTGTTCCCGATCCGTCTGAGCGTTTCCAGCTTAGTCAGGTCGGGATTTTTTGTGCGCC
PEL5       CACGTCACACTGCCAATTGCCGTGGGTAAAATTGTCCGCGCTTAGTAGCAAAATATAATTTAATAACGTAGGTTGTTCCCGATCCGTCTGAGCGTTTCCAGCTTAGTCAGGTCGGGATTTTTTGTGCGCC
LRHMDP2    CACGTAACATTGCCAATTGCCGTGGGTAAAATTGTCCGCGCTTAGTAGCAAAATATAATTTAATAACGTAGGTTGTTCCCGATCTGTCTGAGCGTTTCCAGCTTAGTCAGGTCGGGATTTTTTGTGCGCC
LRHMDP3    CACGTAACATTGCCAATTGCCGTGGGTAAAATTGTCCGCGCTTAGTAGCAAAATATAATTTAATAACGTAGGTTGTTCCCGATCTGTCTGAGCGTTTCCAGCTTAGTCAGGTCGGGATTTTTTGTGCGCC
LMS2-1     CACGTAACATTGCCAATTGCCGTGGGTAAAATTGTCCGCGCTTAGTAGCAAAATATAATTTAATAACGTAGGTTGTTCCCGATCTGTCTGAGCGTTTCCAGCTTAGTCAGATCGGGATTTTTTGTGGTGT
ATCC8530   CACGTAACATTGCCAATTGCCGTGGGTAAAATTGTCCGCGCTTAGTAGCAAAATATAATTTAATAACGTAGGTTGTTCCCGATCTGTCTGAGCGTTTCCAGCTTAGTCAGATCGGGATTTTTTGTGGTGT
LC705      CACGTAACATTGCCAATTGCCGTGGGTAAAATTGTCCGCGCTTAGTAGCAAAATATAATTTAATAACGTAGGTTGTTCCCGATCTGTCTGAGCGTTTCCAGCTTAGTCAGATCGGGATTTTTTGTGGTGT
HN001      CACGTCACACTGCCAATTGCCGTGGGTAAAATTGTCCGCGCTTAGTAGCAAAATATAATTTAATAACGTAGGTTGTTCCCGATCTGTCTGAGCGTTTCCAACTTAGTCAGACCGGGATTTTTTGTGGTGT
E800       CACGTCACACTGCCAATTGCCGTGGGTAAAATTGTCCGCGCTTAGTAGCAAAATATAATTTAATAACGTAGGTTGTTCCCGATCTGTCTGAGCGTTTCCAACTTAGTCAGACCGGGATTTTTTGTGGTGT
R0011      CACGTCACACTGCCAATTGCCGTGGGTAAAATTGTCCGCGCTTAGTAGCAAAATATAATTTAATAACGTGGGTTGTTCCCGATCTGTCTGAGCGTTTCCAGCTTAGTCAGATCGGGATTTTTTGTGGTGT
ATCC21052  CACGTCACACTGCCAATTGCCGTGGGTAAAATTGTCCGCGCTTAGTAGCAAAATATAATTTAATAACGTGGGTTGTTCCCGATCTGTCTGAGCGTTTCCAGCTTAGTCAGATCGGGATTTTTTGTGGTGT
Consensus  CACGTcACAcTGCCAATTGCCGTGGGTAAAATTGTCCGCGCTTAGTAGCAAAATATAATTTAATAACGTaGGTTGTTCCCGATCtGTCTGAGCGTTTCCAgCTTAGTCAGatCGGGATTTTTTGTGgtgt

           391     400       410       420       430       440       450       460       470       480       490       500       510       520
GG         AAATCCTAAAACTTATAAATACTGGAATGATCATTCATATATTCGCTGCTAATTATAATTTAAGAAGTAATATACCGAAGAAAAACTTTATTTATAAAATAACAATTATTATAGTTCCGTTTACGCAGTTC
ATCC53103  AAATCCTAAAACTTATAAATACTGGAATGATCATTCATATATTCGCTGCTAATTATAATTTAAGAAGTAATATACCGAAGAAAAACTTTATTTATAAAATAACAATTATTATAGTTCCGTTTACGCAGTTC
PEL6       AAATCCTAAAACTTATAAATACTGGAATGATCATTCATATATTCGCTGCTAATTATAATTTAAGAAGTAATATACCGAAGAAAAACTTTATTTATAAAATAACAATTATTATAGTTCCGTTTACGCAGTTC
PEL5       AAATCCTAAAACTTATAAATACTGGAATGATCATTCATATATTCGCTGCTAATTATAATTTAAGAAGTAATATACCGAAGAAAAACTTTATTTATAAAATAACAATTATTATAGTTCCGTTTACGCAGTTC
LRHMDP2    AAATCCTAAAACTTATAAATACTGGAATGATCATTCATATATTCGCTGCTAATTATAATTTAAGAAGTAATATACCGAAGAAAAACTTTATTTATAAAATAACAATTATTATAGTTCCGTTTACGCAGTTC
LRHMDP3    AAATCCTAAAACTTATAAATACTGGAATGATCATTCATATATTCGCTGCTAATTATAATTTAAGAAGTAATATACCGAAGAAAAACTTTATTTATAAAATAACAATTATTATAGTTCCGTTTACGCAGTTC
LMS2-1     AAATTCCAATCCCTAGCAATTACTGAATGGTCGTTCATATATTCGCTAATAATTATCATTTAAGAAGTAATATACCGGAGAAAAACTTTATTTATAAAATAACAATTATTATAGTTCCGTTTACGCAGTTC
ATCC8530   AAATTCCAATCCCTAGCAATTACTGAATGGTCGTTCATATATTCGCTAATAATTATCATTTAAGAAGTAATATACCGGAGAAAAACTTTATTTATAAAATAACAATTATTATAGTTCCGTTTACGCAGTTC
LC705      AAATTCCAATCCCTAGCAATTACTGAATGGTCGTTCATATATTCGCTAATAATTATCATTTAAGAAGTAATATACCGGAGAAAAACTTTATTTATAAAATAACAATTATTATAGTTCCGTTTACGCAGTTC
HN001      AAATTCCGATCCCTAGCAATTACTGAATGATCGTTCATATATTCGCTAATAATTATCATTTAAGAAGTAATATACCGGAGAAAAACTTTATTTATAAAATAACAATTATTATAGTTCCGTTTACGCAGTTC
E800       AAATTCCAATCCCTAGCAATTACTGAATGATCGTTCATATATTCGCTAATAATTATCATTTAAGAAGTAATATACCGGAGAAAAACTTTATTTATAAAATAACAATTATTATAGTTCCGTTTACGCAGTTC
R0011      AAATTCCAATCCCTAGCAATTACTGAATGGCCGTTCATATATTCGCTAATAATTATCATTTAAGAAGTAATATACCAGAGAAAAACTTTATTTATAAAATAACAATTATTATAGTTCCGTTTACGCAGTTC
ATCC21052  AAATTCCAATCCCTAGCAATTACTGAATGGCCGTTCATATATTCGCTAATAATTATCATTTAAGAAGTAATATACCAGAGAAAAACTTTATTTATAAAATAACAATTATTATAGTTCCGTTTACGCAGTTC
Consensus  AAATtCcaAtcCcTAgcAATtactGAATGatCgTTCATATATTCGCTaaTAATTATcATTTAAGAAGTAATATACCggAGAAAAACTTTATTTATAAAATAACAATTATTATAGTTCCGTTTACGCAGTTC

           521     530       540       550       560       570       580       590       600       610  615
GG         ATCTTGTTACGCTTAATTCAGCAACAAGTGAAAAATGTAACATGAAAGGAGGCACCCGATTGCCTAGGAAATGGATTCATATGCTGATGTTACTG
ATCC53103  ATCTTGTTACGCTTAATTCAGCAACAAGTGAAAAATGTAACATGAAAGGAGGCACCCGATTGCCTAGGAAATGGATTCATATGCTGATGTTACTG
PEL6       ATCTTGTTACGCTTAATTCAGCAACAAGTGAAAAATGTAACATGAAAGGAGGCACCCGATTGCCTAGGAAATGGATTCATATGCTGATGTTACTG
PEL5       ATCTTGTTACGCTTAATTCAGCAACAAGTGAAAAATGTAACATGAAAGGAGGCACCCGATTGCCTAGGAAATGGATTCATATGCTGATGTTACTG
LRHMDP2    ATCTTGTTACGCTTAATTCAGCAACAAGTGAAAAATGTAACATGAAAGGAGGCACCCGATTGCCTAGGAAATGGATTCATATGCTGATGTTACTG
LRHMDP3    ATCTTGTTACGCTTAATTCAGCAACAAGTGAAAAATGTAACATGAAAGGAGGCACCCGATTGCCTAGGAAATGGATTCATATGCTGATGTTACTG
LMS2-1     ATCTTGTTACGCTTAATTCAGCAACAAGTGAAAAACGTAACATGAAAGGAGGCACCCGATTGCCTAGGAAATGGATTCATATGCTGATGATACTG
ATCC8530   ATCTTGTTACGCTTAATTCAGCAACAAGTGAAAAACGTAACATGAAAGGAGGCACCCGATTGCCTAGGAAATGGATTCATATGCTGATGATACTG
LC705      ATCTTGTTACGCTTAATTCAGCAACAAGTGAAAAATGTAACATGAAAGGAGGCACCCGATTGCCTAGGAAATGGATTCATATGCTGATGATACTG
HN001      ATCTTGTTACGCTTAATTCAGCAACAAGTGAAAAATGTAACATGAAAGGAGGCACCCGATTGCCTAGGAAATGGATTCATATGCTGATGTTACTG
E800       ATCTTGTTACGCTTAATTCAGCAACAAGTGAAAAATGTAACATGAAAGGAGGCACCCGATTGCCTAGGAAATGGATTCATATGCTGATGTTACTG
R0011      ATCTTGTTACGCTTAATTCAGCAACAAGTGAAAAACGTAACATGAAAGGAGGCACCCGATTGCCTAGGAAATGGATTCATATGCTGATGATACTG
ATCC21052  ATCTTGTTACGCTTAATTCAGCAACAAGTGAAAAACGTAACATGAAAGGAGGCACCCGATTGCCTAGGAAATGGATTCATATGCTGATGATACTG
Consensus  ATCTTGTTACGCTTAATTCAGCAACAAGTGAAAAAtGTAACATGAAAGGAGGCACCCGATTGCCTAGGAAATGGATTCATATGCTGATGtTACTG
```
